# Cockroach‐Derived Leucokinin VIII Peptide Accelerates Diabetic Skin Wound Healing by Enhancing Keratinocyte Filopodia Formation

**DOI:** 10.1002/advs.202522333

**Published:** 2026-02-19

**Authors:** Zhengshan Qin, Jinchuan Wu, Xiehua Xiao, Xirui Wang, Nianhui Ding, Xin Zhao, Lu Ma, Jie Li, Lunkun Ma, Cehua Ou, Ning Ma, Jianguo Feng

**Affiliations:** ^1^ Department of Anesthesiology The Affiliated Hospital Southwest Medical University Luzhou Sichuan China; ^2^ Anesthesiology and Critical Care Medicine Key Laboratory of Luzhou The Affiliated Hospital Southwest Medical University Luzhou Sichuan China; ^3^ Department of Pain Management The Affiliated Hospital Southwest Medical University Luzhou Sichuan China; ^4^ Department of Ophthalmology The Affiliated Hospital Southwest Medical University Luzhou Sichuan China; ^5^ School of Clinical Medicine Southwest Medical University Luzhou Sichuan China; ^6^ Nanomedicine Innovation Research and Development Transformation Institute Affiliated Hospital of North Sichuan Medical College Nanchong Sichuan China; ^7^ Inflammation & Allergic Diseases Research Unit The Affiliated Hospital Southwest Medical University Luzhou Sichuan China; ^8^ Medical Electrophysiological Key Laboratory of Sichuan Province Southwest Medical University Luzhou Sichuan China

**Keywords:** diabetic wound healing, FAK‐ACTG1 signaling, filopodia, leucokinin VIII, thermosensitive hydrogel

## Abstract

Cockroach‐derived extracts have shown therapeutic potential for dermatologic and mucosal wound repair. Although recent studies have identified several active components such as exosomes and specific peptides, the therapeutic efficacy and underlying mechanisms of novel short peptides like leucokinin VIII (LK‐VIII) remain to be fully elucidated. Here, we identify LK‐VIII as a potent promoter of keratinocyte migration that effectively alleviates diabetes‐induced migration impairment. Transcriptomic profiling of LK‐VIII‐treated keratinocytes revealed remarkable upregulation of *ACTG1*, encoding γ‐actin. Mechanistically, LK‐VIII activates the FAK‐ACTG1 axis to promote keratinocyte migration and induces filopodia formation, as confirmed by scanning electron microscopy. We then developed an LK‐VIII‐loaded thermosensitive hydrogel system, based on poly(lactide‐co‐glycolide)‐block‐poly(ethylene glycol)‐block‐poly(lactide‐co‐glycolide) (PLGA‐PEG‐PLGA) triblock copolymer, capable of sustained peptide release. In streptozotocin/high fat diet‐induced diabetic mice and db/db mice, hydrogel‐delivered LK‐VIII significantly accelerated cutaneous wound closure. These findings support that the cockroach‐derived LK‐VIII peptide potently accelerates diabetic wound healing via FAK‐ACTG1‐mediated filopodia formation. The novel PLGA‐PEG‐PLGA thermosensitive hydrogel developed in this study represents a promising therapeutic approach for sustained peptide delivery.

## Graphical abstract

This study identifies the cockroach‐derived peptide leucokinin VIII as a potent accelerator of diabetic wound healing. By activating the FAK‐ACTG1 axis, the peptide drives keratinocyte migration and filopodia formation. Delivered via a developed PLGA‐PEG‐PLGA thermosensitive hydrogel for sustained release, this approach significantly hastens wound closure in diabetic mice, presenting a promising therapeutic strategy.

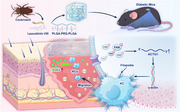

## 1 Introduction

Diabetes mellitus is a global health challenge affecting millions of people and poses a significant socioeconomic burden. According to the International Diabetes Federation, 589 million adults were estimated to have diabetes in 2024, and this number is projected to increase by 45% to 853 million by 2050 [1]. Impaired wound healing is one of the most challenging complications for approximately 15% of diabetic patients, even with conventional clinical interventions that focus on glycemic control, debridement, and infection management [2]. This therapeutic gap underscores the urgent need for innovative treatment strategies and advanced drug delivery systems.

The skin, the largest organ of the human body, provides a protective barrier against biological and chemical agents while regulating body temperature and maintaining fluid balance [3]. Cutaneous wound healing encompasses four overlapping phases: hemostasis, inflammation, proliferation, and remodeling, which are orchestrated by complex cellular and molecular mechanisms. However, the diabetic microenvironment severely disrupts this highly coordinated process through multiple pathological alterations, including enhanced oxidative stress, dysregulated inflammatory responses, accumulation of advanced glycation end products, impaired angiogenesis, and compromised re‐epithelialization [4].

Re‐epithelialization represents a critical determinant of wound closure and the restoration of skin integrity and barrier function. This process primarily relies on keratinocytes at the wound margins, which coordinate proliferation, migration, and differentiation during tissue repair [5]. Under diabetic conditions, the biological functions of keratinocytes are significantly impaired, characterized by reduced cell viability, diminished proliferative capacity, increased apoptosis, and compromised migration and differentiation [6]. Directional keratinocyte migration requires dynamic remodeling of cell adhesion complexes and cytoskeletal structures. In particular, filopodia have been previously believed to function primarily as guiding cues, utilizing their tip complexes to facilitate cellular elongation [7]; however, these processes are significantly impaired in the diabetic pathological microenvironment [8]. Therefore, the targeted modulation of cytoskeletal remodeling and filopodia formation is a promising therapeutic strategy for diabetic wound healing.

Recent studies have highlighted the potential of bioactive peptides derived from various animal sources in promoting tissue regeneration [9, 10]. For instance, the peptide Cy _RL‐QN15_, a novel ultra‐short cyclic heptapeptide, was recently shown to promote skin repair, diabetic wound healing [11], follicle neogenesis [12], and mucosal repair [13]. Furthermore, cyclic heptapeptide FZ1, which acts as an integrin αvβ3 agonist, exhibited significantly greater efficacy than rh‐bFGF and Cy _RL‐QN15_ in promoting skin cell proliferation and migration [14]. *Kangfuxin*, an extract of *Periplaneta americana* widely used in clinical practice especially in China, has demonstrated notable efficacy in mucosal and cutaneous wound healing [15, 16]. Several studies have primarily attributed its therapeutic effects to small chemical molecules isolated from the extract, identifying these low‐molecular weight constituents as key drivers of tissue repair [17, 18]. In addition, bioactive peptides offer remarkable advantages for wound repair because of their strong penetration ability, minimal immunogenicity, and high targeting specificity [19]. However, investigations into the functional roles of bioactive peptides derived from *P. americana* extracts are relatively limited. For example, leucokinin VIII (LK‐VIII), a bioactive peptide isolated from *P. americana*, was previously reported to regulate neuronal activity, diuresis, and ion signaling pathways [20, 21]. However, its effects on epidermal keratinocytes and its potential role in wound repair remain unexplored.

Hydrogels have emerged as promising carriers for wound healing applications due to their exceptional biocompatibility, plasticity, and controlled‐release properties [22]. In particular, thermosensitive hydrogels respond to temperature changes by undergoing sol‐gel phase transitions at specific temperatures, which offers innovative approaches for precise drug delivery and controlled release systems [23]. Among these, poly(lactide‐co‐glycolide)‐block‐poly(ethylene glycol)‐block‐poly(lactide‐co‐glycolide) (PLGA‐PEG‐PLGA) triblock copolymer hydrogels demonstrate superior injectability, controlled degradability, moisturizing properties, transparency, and sustained drug release characteristics [24, 25], providing distinct advantages for diabetic wound management. Nevertheless, optimizing drug loading efficiency and release kinetics for precise modulation of the wound healing process remains a challenge.

In this study, we demonstrate that the cockroach‐derived LK‐VIII peptide enhances keratinocyte γ‐actin synthesis through *ACTG1* upregulation, promoting cell adhesion and cytoskeletal remodeling that facilitates the directional migration of keratinocytes at wound margins, effectively accelerating re‐epithelialization in diabetic wounds. To enable clinical translation, we successfully developed an LK‐VIII‐loaded PLGA‐PEG‐PLGA thermosensitive hydrogel system that rapidly forms a gel at body temperature and allows for sustained and controlled release of the bioactive peptide, significantly improving the healing efficiency of diabetic wounds. This integrated approach, which combines molecular targeting mechanisms with advanced biomaterial design, provides a novel therapeutic strategy for diabetic wound healing with significant clinical application potential.

## 2 Results

### 2.1 LK‐VIII Promotes Keratinocyte Migration

**FIGURE 1:**
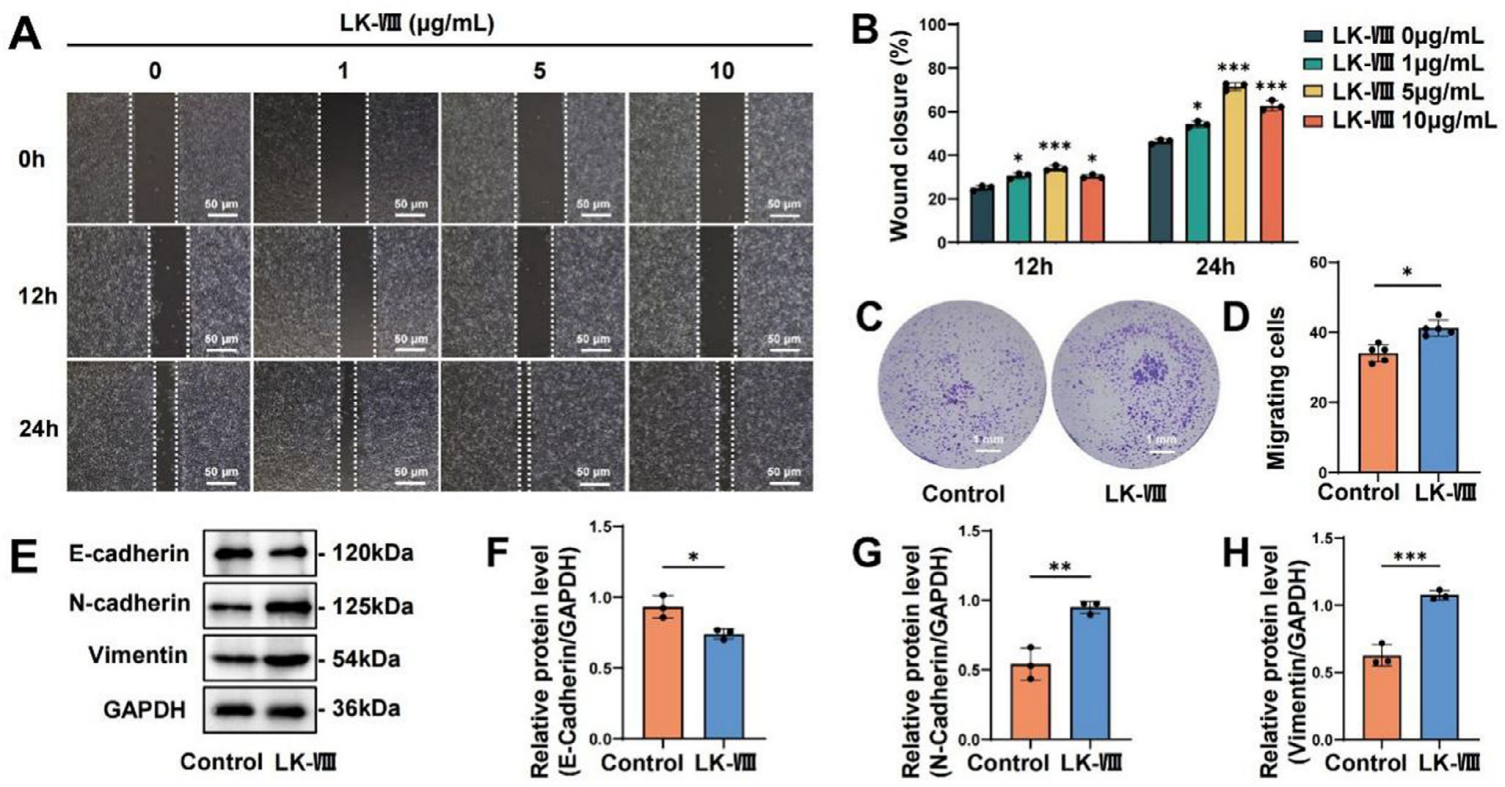
LK‐VIII promoted HaCaT cell migration. (A) The effects of different LK‐VIII concentrations (0, 1, 5, and 10 µg/mL) on HaCaT cell migration were examined using scratch wound healing assays at 0, 12, and 24 h. (B) Quantification of wound closure percentage. (C) Transwell migration assay was used to demonstrate the effect of LK‐VIII (5 µg/mL) on HaCaT cell migration. (D) Quantification of migrated cells in five randomly selected microscopic fields (100× magnification). (E) The protein levels of E‐cadherin, N‐cadherin and Vimentin were examined by Western blot in HaCaT cells after 24 h of treatment with LK‐VIII (5 µg/mL). GAPDH served as a loading control. (F) Densitometric quantification of E‐cadherin protein levels normalized to those of GAPDH. Data are presented as mean ± SEM (B, D, F). Data among multiple groups were compared by one‐way ANOVA test, followed by Tukey post hoc test (B); data between two groups were compared by independent‐sample two‐tailed Student's t‐test (D, F, G, H); *p < 0.05, **p < 0.01, ***p < 0.001 versus control group.

Keratinocyte migration is fundamental to wound re‐epithelialization during healing [5]. We initially assessed the cytotoxicity of LK‐VIII on human immortalized keratinocytes (HaCaT) using the Cell Counting Kit‐8 (CCK‐8) assay. Various concentrations (0, 1, 5, and 10 µg/mL) maintained cell viability above 100% after 24 h exposure, confirming the safety of the selected concentration range, while no significant increase in cell viability was observed upon exposure to 1, 5, or 10 µg/mL (Figure S1A). Subsequently, we investigated the effects of different LK‐VIII concentrations on HaCaT cells migration. Scratch assay results demonstrated that LK‐VIII‐treated groups exhibited enhanced wound closure capacity at both 12 h and 24 h, with the 5 µg/mL dose showing the most significant effect (Figure 1A,B). Based on these findings, 5 µg/mL was selected as the optimal concentration for subsequent experiments. To further validate the pro‐migratory effect, we performed Transwell migration assays, which confirmed that 5 µg/mL LK‐VIII significantly promoted HaCaT cells migration (Figure 1C,D). To explore the underlying mechanisms, we examined the levels of E‐cadherin, a key protein involved in the regulation of cell migration. Western blot analysis revealed that LK‐VIII treatment significantly downregulated E‐cadherin levels and increased the protein levels of Vimentin and N‐cadherin (Figure 1E–H). Normal Human Epidermal Keratinocytes (NHEKs), purchased from Shanghai QuiCell Biotechnology, were also treated with LK‐VIII peptide to assess its effects. The treatment of NHEKs with the peptide demonstrated similar effects to those observed in HaCaT cells (Figure S1B–I). These results demonstrate that LK‐VIII promotes keratinocyte migration, rather than enhancing cell proliferation.

### 2.2 LK‐VIII Ameliorates AGEs‐Induced Dysfunction in HaCaT Cells

**FIGURE 2:**
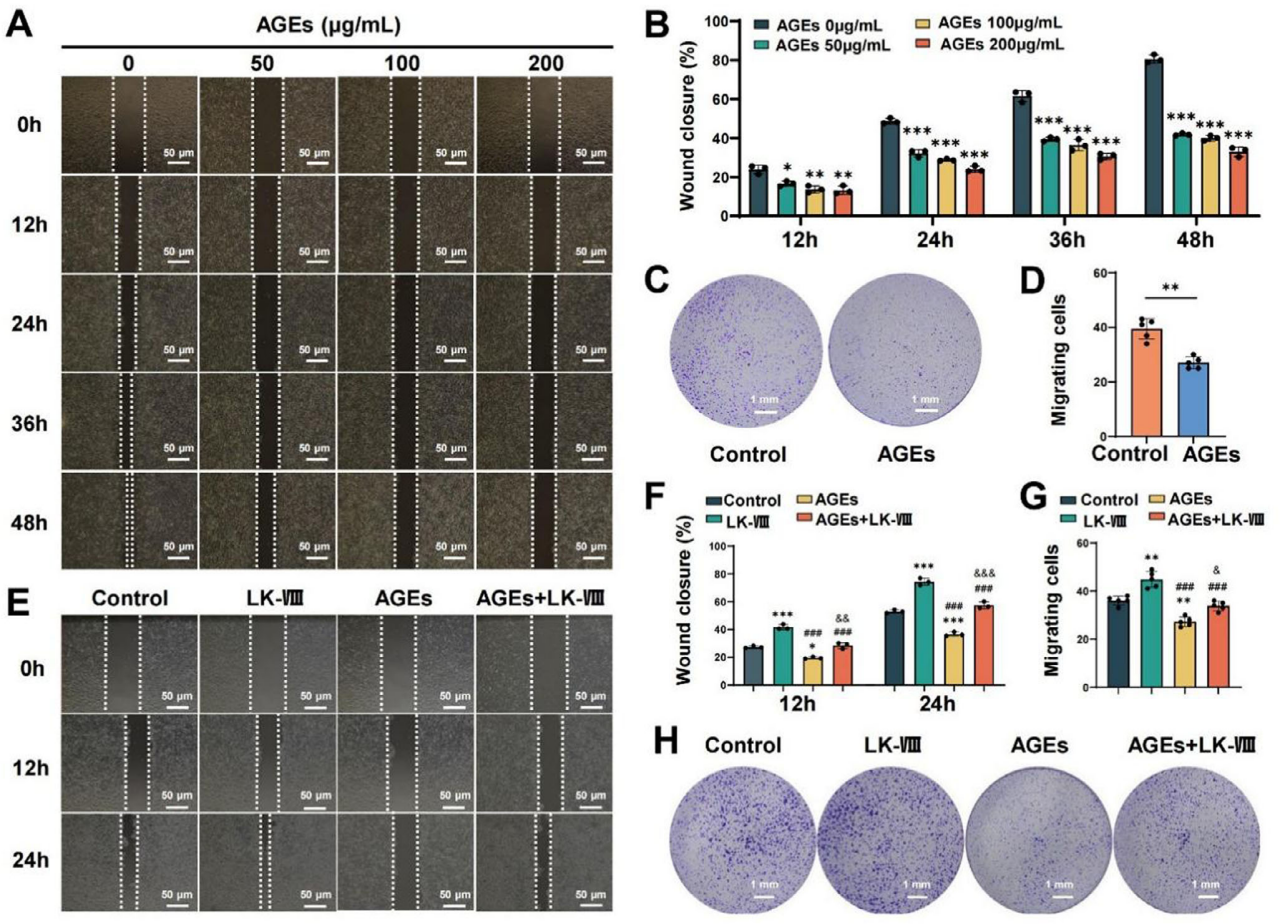
LK‐VIII ameliorated AGEs‐induced migration impairment in HaCaT cells. (A) Scratch wound healing assays were used to evaluate the dose‐dependent inhibitory effects of AGEs (0, 50, 100, and 200 µg/mL) on HaCaT cell migration over 48 h. (B) Quantification of wound closure percentage at indicated time points. (C) The effects of AGEs (100 µg/mL) on HaCaT cell migration were examined using Transwell migration assays. (D) Quantification of migrated cells from five randomly selected fields. (E) Scratch assays were performed to evaluate the protective effects of LK‐VIII (5 µg/mL) against AGEs (100 µg/mL)‐induced inhibition of cell migration over 24 h. (F) Quantification of wound closure percentage at 12 and 24 h. (G) Quantification of migrated cells in Transwell assays for all treatment groups. (H) Transwell migration assays showing cell migration in groups treated with LK‐VIII, AGEs, or their combination. Data are presented as mean ± SEM (B, D, F, G). Data among multiple groups were compared by one‐way ANOVA test, followed by Tukey post hoc test (B, F, G). Data between two groups were compared by independent‐sample two‐tailed Student's t‐test (D). *p < 0.05, **p < 0.01, ***p < 0.001 versus control group; ^###^ *p* < 0.001 versus LK‐VIII group; ^&^ *p* < 0.05, ^&&^ *p* < 0.01, ^&&&^ *p* < 0.001 versus AGEs group.

Advanced glycation end products (AGEs) accumulation is a key pathological factor in delayed diabetic wound healing [26]. Scratch assay showed that AGEs treatment (50, 100, and 200 µg/mL) significantly prolonged wound closure time in a dose‐dependent manner (Figure 2A,B). Transwell migration assays confirmed that 100 µg/mL AGEs significantly reduced HaCaT cell migration capacity compared to untreated controls (Figure 2C,D). These findings indicate that AGEs impair keratinocyte migration, contributing to delayed wound healing under diabetic conditions.

To investigate the therapeutic potential of LK‐VIII in diabetic wound healing, we examined its ability to counteract AGEs‐induced cellular dysfunction. Scratch assays revealed that LK‐VIII (5 µg/mL) promoted wound closure under normal conditions and significantly ameliorated AGEs (100 µg/mL)‐mediated migration impairment at both 12 and 24 h time points (Figure 2E,F). The protective effect of LK‐VIII was further validated by Transwell migration assays, which showed that LK‐VIII treatment effectively restored cell migration capacity in AGEs‐treated cells, with migrating cell numbers approaching those of untreated controls (Figure 2G,H). Given that the diabetic wound microenvironment is marked by notably elevated reactive oxygen species (ROS) levels, which can be triggered by the accumulation of AGEs, we assessed the protective effects of LK‐VIII against H_2_O_2_‐induced oxidative stress. The findings indicated that LK‐VIII (5 µg/mL) also efficiently alleviated H_2_O_2_‐mediated migration impairment at both 12 and 24 h, as verified by scratch and transwell assays (Figure S2).

Collectively, these results demonstrate that LK‐VIII can counteract AGEs or ROS ‐induced migration dysfunction in keratinocytes, supporting its potential therapeutic application in diabetic wound healing.

### 2.3 Transcriptomic Profiling Reveals *ACTG1* as a Key Effector of LK‐VIII‐Mediated Cellular Response

**FIGURE 3:**
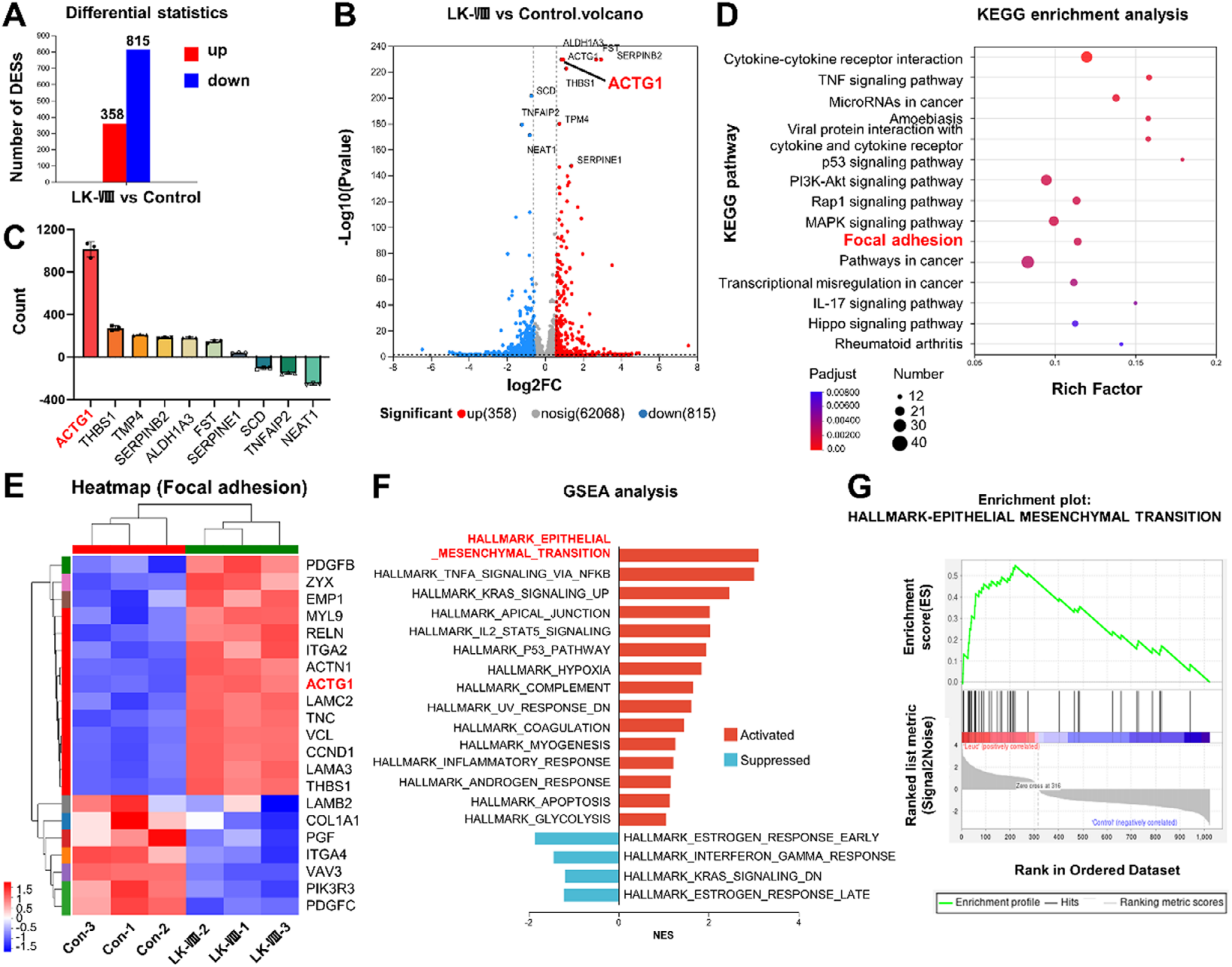
Transcriptomic analysis revealed *ACTG1* as a key mediator of LK‐VIII‐promoted cell migration. (A) Bar chart showing the number of differentially expressed genes (DEGs) following LK‐VIII treatment. (B) Volcano plot displaying the distribution of DEGs, with the top 10 most significantly changed genes labeled (red: upregulated; blue: downregulated; gray: non‐significant). (C) Expression count changes of the top 10 DEGs. (D) KEGG pathway enrichment analysis presented as a bubble plot, where bubble size represents gene count and color intensity indicates statistical significance. (E) Heatmap of focal adhesion pathway‐related genes across experimental replicates (red, upregulation; blue, downregulation). *ACTG1* (in red) shows consistent upregulation across all replicates. (F) Gene set enrichment analysis (GSEA) results showing significantly enriched gene sets (red, activated pathways; blue, suppressed pathways), with epithelial‐mesenchymal transition (EMT) displaying the highest enrichment score. (G) Enrichment plot for the EMT gene set showing the enrichment score curve (top), gene hit positions (middle), and ranking metric (bottom).

To elucidate the molecular mechanisms underlying LK‐VIII‐promoted cell migration, we performed transcriptomic analysis comparing LK‐VIII‐treated and control HaCaT cells. RNA sequencing identified 1,173 differentially expressed genes (DEGs), with 358 upregulated and 815 downregulated genes (Figure 3A). Volcano plot analysis revealed the global distribution of gene expression changes and highlighted the top 10 most significantly altered genes (Figure 3B and Table 1). Notably, the γ‐actin encoding gene *ACTG1* exhibited the most substantial increase in expression (Figure 3C). Although *ACTG1* did not display the highest fold change, its elevated baseline expression and significant upregulation suggested its potential importance in mediating LK‐VIII effects. KEGG pathway enrichment analysis demonstrated that the focal adhesion pathway was significantly enriched among the upregulated genes (Figure 3D). Moreover, heatmap visualization of focal adhesion‐related genes showed that *ACTG1* was consistently upregulated following LK‐VIII treatment (Figure 3E). Gene Set Enrichment Analysis (GSEA) using hallmark gene sets revealed that the epithelial‐mesenchymal transition (EMT) was the most significantly enriched pathway (Figure 3F), with the enrichment profile demonstrating coordinated activation of EMT‐associated genes (Figure 3G).

**TABLE 1:** Top 10 most significantly altered genes following LK‐VIII treatment.

Gene name	FC(LK‐VIII/Control)	Log_2_FC(LK‐VIII/Control)	*p* value
*FST*	6.429563207	2.684720731	0
*ALDH1A3*	1.928478968	0.947463412	0
*SERPINB2*	7.701237884	2.945090361	0
*ACTG1*	1.824043481	0.867140121	2.50E‐230
*THBS1*	2.166656802	1.115470649	2.43E‐223
*SCD*	0.607997044	−0.717863785	4.08E‐202
*TPM4*	1.688413791	0.755668519	1.14E‐180
*TNFAIP2*	0.430357366	−1.216392932	7.59E‐180
*NEAT1*	0.573839068	−0.801281902	8.56E‐172
*SERPINE1*	2.607969999	1.382927273	4.50E‐148

To further understand the functional significance of *ACTG1* in the cellular response to LK‐VIII, we constructed a protein‐protein interaction (PPI) network of DEG‐encoded proteins. ACTG1 occupied a central position within the network, displaying the highest degree of connectivity (10 interactions out of 252 total proteins) and serving as a hub node (Figure S3A). Its primary interaction partners included cytoskeletal proteins (VCL and ACTN1), myosin components (MYH9 and MYL9), and other regulators of cell adhesion and migration (EZR, ZYX, BAIAP2, SYNPO, AFDN, and THRA) (Table 2). Furthermore, Gene Ontology (GO) enrichment analysis revealed significant enrichment of biological processes related to cellular response regulation and signal transduction (Figure S3B). Reactome pathway enrichment analysis (Figure S3C) identified immune‐inflammatory pathways, such as interleukin signaling, interferon‐gamma signaling, and the immune system pathway, as well as transcriptional regulatory pathways, such as FOXO and NOTCH3, which are closely associated with skin wound healing.

**TABLE 2:** Major interacting proteins of ACTG1 and their interaction characteristics.

Node1	Node2	Node1 accession id	Node2 accession id	Combined score	Coexpression	Experimentally determined interaction
ACTG1	VCL	ENSG00000184009	ENSG00000035403	0.99	0.056	0.05
ACTG1	MYH9	ENSG00000184009	ENSG00000100345	0.978	0.084	0.238
ACTG1	ACTN1	ENSG00000184009	ENSG00000072110	0.972	0.07	0.125
ACTG1	EZR	ENSG00000184009	ENSG00000092820	0.962	0.067	0.054
ACTG1	MYL9	ENSG00000184009	ENSG00000101335	0.94	0.11	0.126
ACTG1	ZYX	ENSG00000184009	ENSG00000159840	0.931	0	0
ACTG1	BAIAP2	ENSG00000184009	ENSG00000175866	0.923	0	0.051
ACTG1	SYNPO	ENSG00000184009	ENSG00000171992	0.92	0.054	0.144
ACTG1	AFDN	ENSG00000184009	ENSG00000130396	0.915	0	0
ACTG1	THRA	ENSG00000184009	ENSG00000126351	0.903	0	0.047

Collectively, these transcriptomic findings identify *ACTG1* as a key molecular effector of LK‐VIII action and implicate focal adhesion remodeling and EMT pathway activation as primary mechanisms underlying enhanced keratinocyte migration.

### 2.4 *ACTG1*‐Mediated Cytoskeletal Remodeling Underlies LK‐VIII‐Promoted Keratinocyte Migration

**FIGURE 4:**
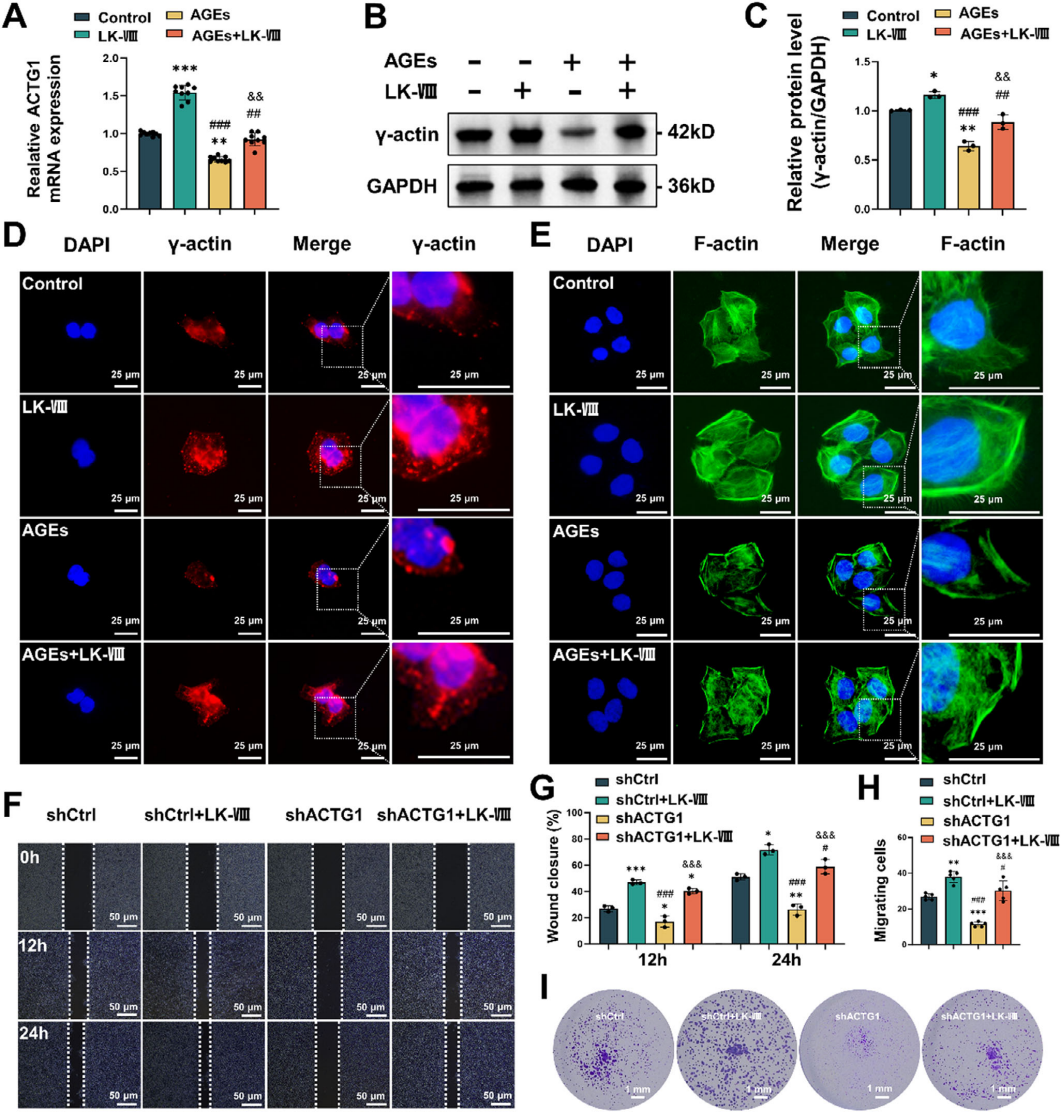
The involvement of *ACTG1* in cytoskeletal remodeling underlying LK‐VIII‐promoted keratinocyte migration. (A) The levels of *ACTG1* mRNA were tested by quantitative PCR at 24 h following AGEs and/or LK‐VIII treatments. Data were normalized to GAPDH and presented as fold change relative to control. (B) Western blot analysis of γ‐actin (encoded by *ACTG1*) protein levels at 24 h following treatment with AGEs and/or LK‐VIII. GAPDH served as a loading control. (C) Densitometric quantification of γ‐actin protein levels normalized to GAPDH. Data are presented as mean ± SEM (A, C), and statistical significance was determined using one‐way ANOVA followed by Tukey's post hoc test (A, C). *p < 0.05, **p < 0.01, ***p < 0.001 versus control group; ^##^ *p* < 0.01, ^###^ *p* < 0.001 versus LK‐VIII group; ^&&^ *p* < 0.01 versus AGEs group. (D) Immunofluorescence staining showing the levels ssion of γ‐actin (red) in HaCaT cells following AGEs and/or LK‐VIII treatments, with DAPI (blue) for nuclear staining. (E) Phalloidin staining of F‐actin cytoskeleton (green) with nuclear counterstaining (DAPI, blue). (F) Scratch wound healing assays were performed to assess cell migration at 12 and 24 h in control (shCtrl) and *ACTG1*‐knockdown (sh*ACTG1*) cells with or without LK‐VIII treatment. (G) Quantitative analysis of wound closure percentage at 12 and 24 h. (H) Quantification of migrated cells in Transwell assays. (I) Representative Transwell migration images showing crystal violet‐stained cells. Data are presented as mean ± SEM (G, H). Statistical significance was determined using one‐way ANOVA followed by Tukey's post hoc test (G, H).* *p* < 0.05, **p < 0.01, ***p < 0.001 versus shCtrl; ^#^ *p* < 0.05, ^###^ *p* < 0.001 versus shCtrl+LK‐VIII; ^&&&^ *p* < 0.001 versus sh*ACTG1*.

Based on transcriptomic analysis identifying *ACTG1* as a key target, we validated its role in LK‐VIII‐mediated cellular responses. Quantitative PCR confirmed that LK‐VIII treatment significantly upregulated *ACTG1* mRNA expression, while AGEs treatment reduced it. Importantly, LK‐VIII co‐treatment effectively reversed AGEs‐induced *ACTG1* downregulation (Figure 4A). Western blot analysis further validated these findings at the protein level, demonstrating corresponding changes in γ‐actin protein abundance (Figure 4B,C).

To examine the functional consequences of altered *ACTG1* expression, we performed immunofluorescence analysis of γ‐actin and F‐actin organization. LK‐VIII treatment enhanced γ‐actin fluorescence intensity with organized cytoplasmic distribution, while AGEs treatment reduced γ‐actin expression with disrupted localization patterns. LK‐VIII co‐treatment restored normal γ‐actin distribution in AGEs‐challenged cells (Figure 4D). Phalloidin staining revealed that LK‐VIII promoted well‐organized F‐actin fiber formation, whereas AGEs induced cytoskeletal disassembly and loss of organized filamentous structures. LK‐VIII co‐treatment preserved cytoskeletal integrity and prevented AGEs‐mediated F‐actin disruption (Figure 4E).

To investigate the role of *ACTG1* in LK‐VIII‐mediated migration enhancement, we generated *ACTG1*‐knockdown HaCaT cells by transient transfection of specific *ACTG1* shRNAs. Scratch wound healing assays demonstrated that *ACTG1* silencing significantly impaired wound closure compared to that in control cells (shCtrl). While LK‐VIII treatment enhanced migration in control cells, its effect was substantially diminished in *ACTG1*‐silenced cells (Figure 4F,G). In addition, Transwell migration assays indicated that *ACTG1* knockdown reduced migration capacity and attenuated LK‐VIII‐mediated migration enhancement (Figure 4H,I).

These results demonstrate that LK‐VIII promotes keratinocyte migration through *ACTG1*‐mediated cytoskeletal remodeling and support *ACTG1* as a critical mediator of LK‐VIII's beneficial effects in diabetic wound healing.

### 2.5 LK‐VIII Promotes Cellular Filopodia Formation

**FIGURE 5:**
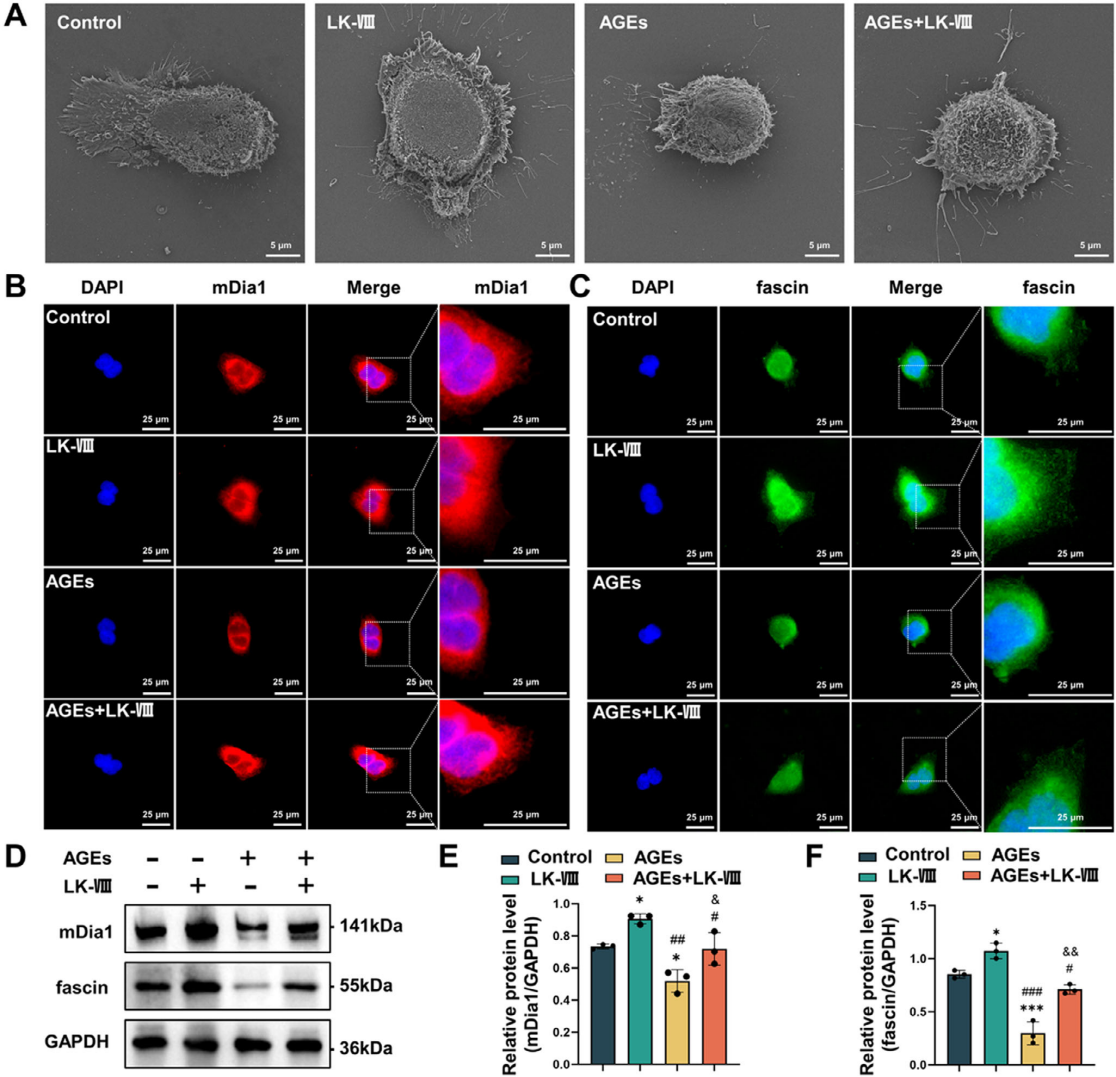
The effects of LK‐VIII on filopodia formation in keratinocytes. (A) Scanning electron microscopy images showing cellular morphology and filopodia in HaCaT cells at 24 h following AGEs and/or LK‐VIII treatments. (B) Immunofluorescence staining of mDia1 (red) and nuclei (DAPI, blue). (C) Immunofluorescence staining of fascin (green) and nuclei (DAPI, blue). (D) Western blot analysis of mDia1 and fascin protein levels at 24 h following treatment with AGEs and/or LK‐VIII. GAPDH served as loading control. (E, F) Densitometric quantification of mDia1 (E) and fascin (F), protein levels normalized to GAPDH. Data are presented as mean ± SEM. Statistical significance was determined using one‐way ANOVA followed by Tukey's post hoc test (E, F); *p < 0.05, ***p < 0.001 versus control group; ^#^ *p* < 0.05, ^##^ *p* < 0.01, ^###^ *p* < 0.001 versus LK‐VIII group; ^&^ *p* < 0.05, ^&&^ *p* < 0.01 versus AGEs group.

To investigate the molecular structural basis underlying LK‐VIII‐enhanced keratinocyte migration, we examined cellular morphology and key actin regulatory proteins. Scanning electron microscopy analysis revealed that LK‐VIII treatment markedly enhanced filopodia formation compared to control cells (Figure 5A). Treatment with AGEs severely impaired protrusion formation, resulting in rounded cellular morphology with minimal membrane extensions. Notably, LK‐VIII exposure effectively reversed the inhibitory effects of AGEs, restoring protrusion formation in LK‐VIII‐treated cells.

We subsequently investigated the influence of LK‐VIII on the expression of mDia1 and fascin, two critical proteins involved in filopodia formation. Immunostaining showed that LK‐VIII treatment significantly increased mDia1 level and promoted its localization to the cell cortex and extending protrusions (Figure 5B). Similarly, LK‐VIII elevated fascin level and enhanced its accumulation in filopodial structures (Figure 5C). In contrast, AGEs treatment reduced the level of both proteins and disrupted their subcellular localization, whereas co‐treatment with LK‐VIII restored their distribution to nearly normal patterns. Consistent with these observations, western blot analysis showed that LK‐VIII upregulated mDia1 and fascin protein level, while AGEs reduced both proteins, and LK‐VIII co‐treatment effectively reversed these reductions (Figure 5D–F).

These results indicate that LK‐VIII enhances keratinocyte migration by promoting filopodia formation through the upregulation of the cytoskeletal regulators mDia1 and fascin, thereby generating functional protrusions necessary for efficient cell movement.

### 2.6 LK‐VIII Promotes Keratinocyte Migration via FAK‐ACTG1 Signaling Axis

As described above, LK‐VIII treatment upregulated genes in keratinocytes that are significantly enriched in the focal adhesion pathway (Figure 3D), in which focal adhesion kinase (FAK) is phosphorylated and activated, regulating cytoskeletal remodeling and cell motility. Therefore, we hypothesized that FAK activation is an upstream mechanism mediating LK‐VIII‐induced *ACTG1* expression and cell migration.

**FIGURE 6:**
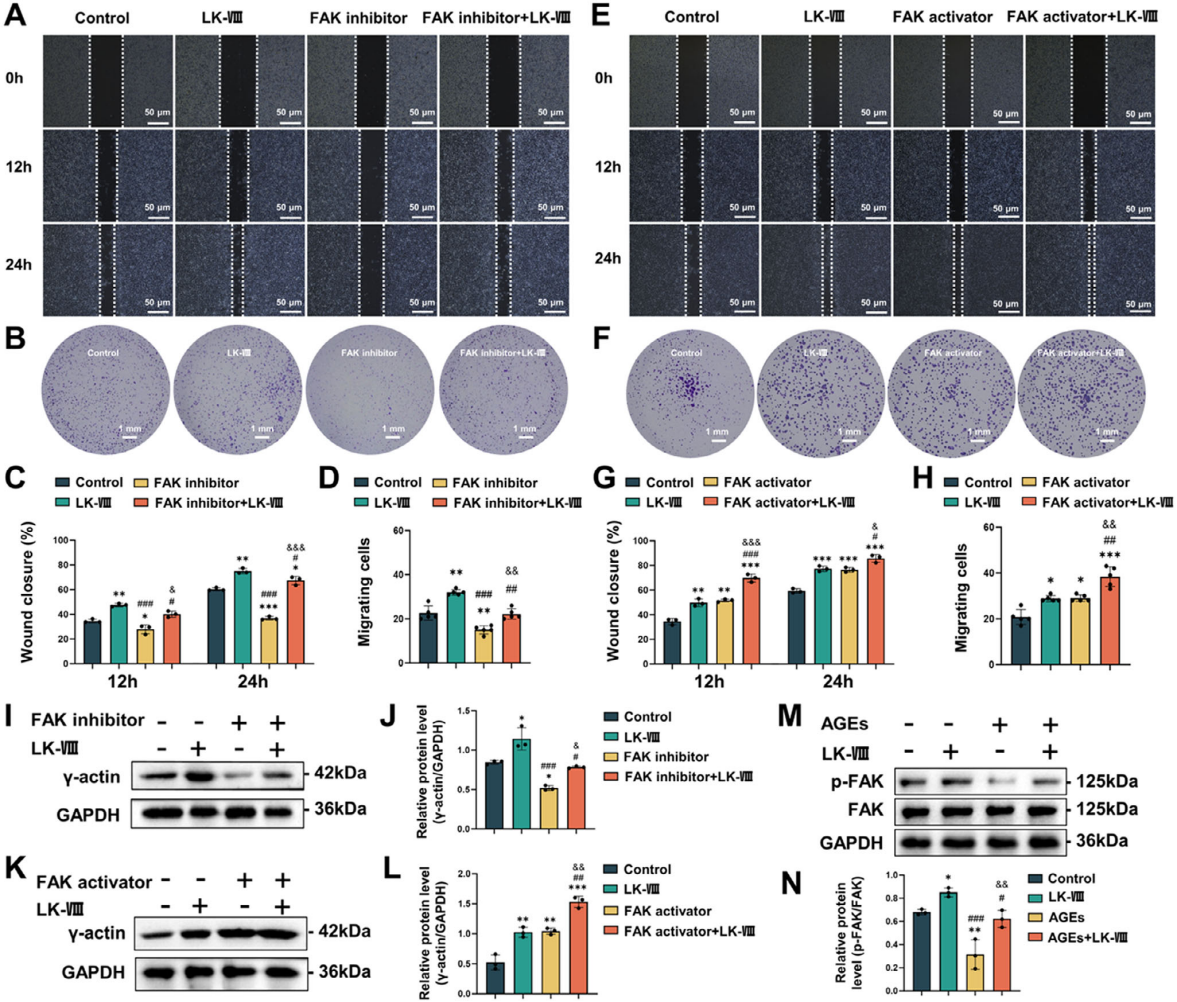
LK‐VIII regulated keratinocyte migration through the FAK‐ACTG1 signaling pathway. (A) Scratch wound healing assays in HaCaT cells at 0, 12, and 24 h with LK‐VIII (5 µg/mL) and/or FAK inhibitor (10 µM). (B) Transwell migration assays showing migrated cells stained with crystal violet at 24 h. (C) Quantitative analysis of wound closure percentage at 12 and 24 h for FAK inhibitor experiments. (D) Quantification of migrated cells per microscopic field for FAK inhibitor experiments. Data are presented as mean ± SEM (C, D), and statistical significance was determined using one‐way ANOVA followed by Tukey's post hoc test(C, D).*p< 0.05, **p < 0.01, ***p < 0.001 versus control group; ^#^ *p* < 0.05, ^##^ *p* < 0.01, ^###^ *p* < 0.001 versus LK‐VIII group; ^&^ *p* < 0.05, ^&&^ *p* < 0.01, ^&&&^ *p* < 0.001 versus FAK inhibitor group. (E) Scratch wound healing assays at 0, 12 and 24 h with LK‐VIII (5 µg/mL) and/or FAK activator (10 nM). (F) Transwell migration assays at 24 h. (G) Quantification of wound closure percentage at 12 and 24 h. (H) Quantification of migrated cells per field. Data are presented as mean ± SEM (G, H). Statistical significance was determined using one‐way ANOVA followed by Tukey's post hoc test(G, H). *p < 0.05, **p < 0.01, ***p < 0.001 versus control group; ^#^ *p* < 0.05, ^##^ *p* < 0.01, ^###^ *p* < 0.001 versus LK‐VIII group; ^&^ *p* < 0.05, ^&&^ *p* < 0.01, ^&&&^ *p* < 0.001 versus FAK activator group. (I) Western blot analysis of γ‐actin levels at 24 h with FAK inhibitor. GAPDH served as a loading control. (J) Densitometric quantification of γ‐actin protein levels normalized to GAPDH. Data are presented as mean ± SEM. Statistical significance was determined using one‐way ANOVA followed by Tukey's post hoc test. *p < 0.05 versus control group; ^#^ *p* < 0.05, ^###^ *p* < 0.001 versus LK‐VIII group; ^&^ *p* < 0.05 versus FAK inhibitor group. (K) Western blot analysis of γ‐actin levels at 24 h with FAK activator. GAPDH served as a loading control. (L) Densitometric quantification of γ‐actin protein levels normalized to GAPDH. Data are presented as mean ± SEM, and statistical significance was determined using one‐way ANOVA followed by Tukey's post hoc test. **p < 0.01, ***p < 0.001 versus control group; ^##^ *p* < 0.01 versus LK‐VIII group; ^&&^ *p* < 0.01 versus FAK activator group. (M) Western blot of p‐FAK and total FAK at 24 h with AGEs (100 µg/mL) and/or LK‐VIII (5 µg/mL); GAPDH served as loading control. (N) Quantification of p‐FAK/total FAK ratio. Data are presented as mean ± SEM. Statistical significance was determined using one‐way ANOVA followed by Tukey's post hoc test. *p < 0.05, **p < 0.01 versus control group; ^#^ *p* < 0.05, ^###^ *p* < 0.001 versus LK‐VIII group; ^&&^ *p* < 0.01 versus AGEs group.

Functional analysis using the FAK inhibitor PF‐573228 (10 µm) markedly reduced keratinocyte migration in both scratch wound healing and Transwell assays, whereas co‐treatment with LK‐VIII partially restored migratory capacity (Figure 6A–D). Conversely, combined treatment with the FAK activator ZINC40099027 (10 nM) and LK‐VIII exhibited a synergistic enhancement of cell migration, significantly accelerating wound closure compared with either treatment alone (Figure 6E–H). At the molecular level, FAK inhibition significantly downregulated γ‐actin protein level, which was reversed by LK‐VIII (Figure 6I,J). The FAK activator combined with LK‐VIII further increased γ‐actin level, confirming a functional relationship between FAK activation and *ACTG1* regulation (Figure 6K,L). Given that the accumulation of AGEs in patients with diabetes impairs wound healing, we evaluated FAK signaling under diabetic‐mimicking conditions. AGEs treatment significantly reduced FAK phosphorylation level, consistent with diminished cellular migration in diabetic wounds. Importantly, LK‐VIII restored p‐FAK level and rescued AGEs‐induced migration defects (Figure 6M,N). These results indicate that FAK‐ACTG1 signaling mediates the protective effect of LK‐VIII against diabetes‐induced impairment of keratinocyte migration.

### 2.7 Development and Characterization of LK‐VIII‐Loaded Thermosensitive Hydrogel for Sustained Drug Delivery

**FIGURE 7:**
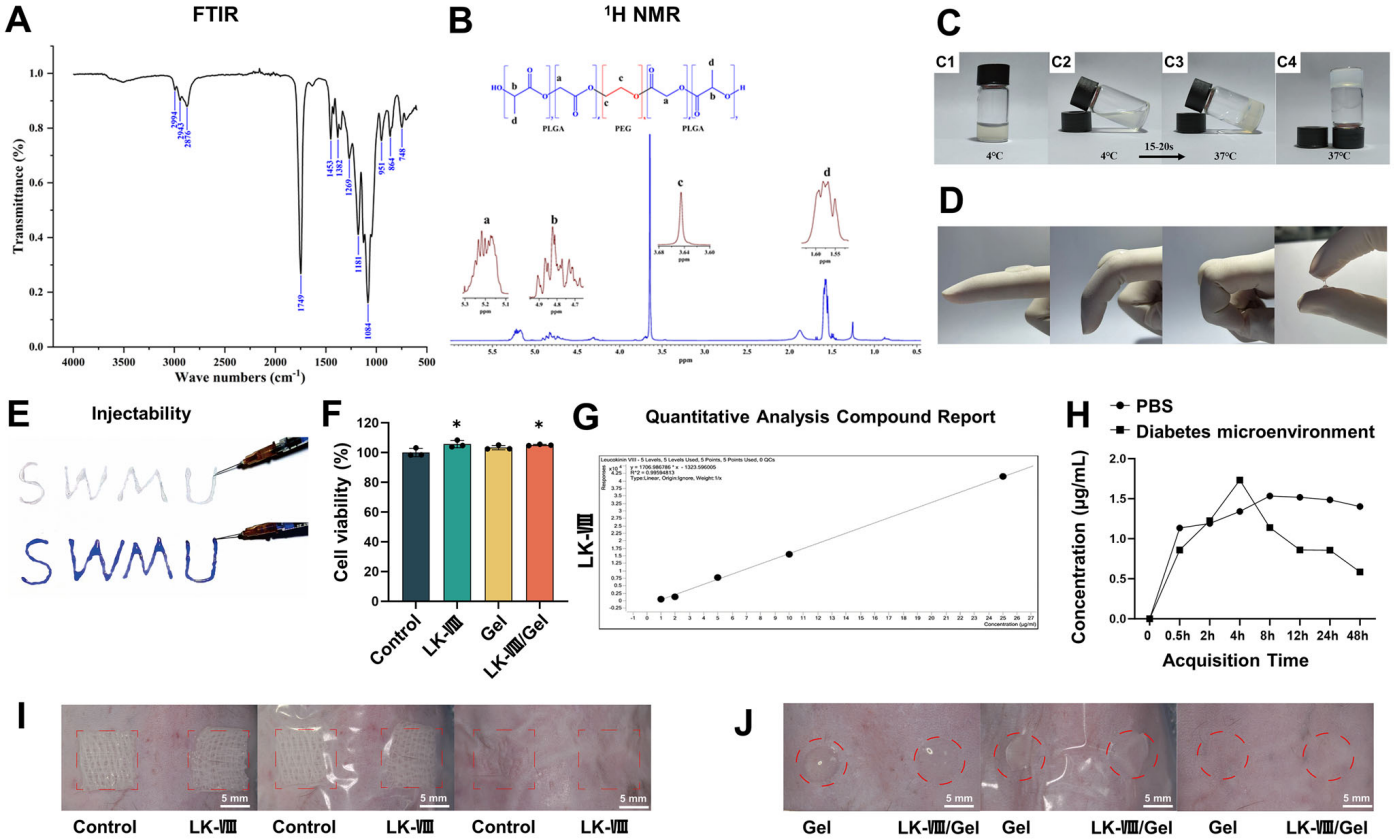
Synthesis, characterization, and biocompatibility assessment of LK‐VIII‐loaded thermosensitive hydrogel. (A) FTIR spectrum of PLGA‐PEG‐PLGA triblock copolymer showing characteristic absorption peaks. (B) ^1^H NMR spectrum of the synthesized triblock copolymer with chemical shift assignments. (C) Temperature‐responsive sol‐gel phase transition behavior at 4°C (panels C1 and C2) and 37°C (panels C3 and C4). (D) Mechanical properties and adhesion assessment on curved surfaces. (E) Injectability test through a 27‐gauge syringe. (F) Cell viability of HaCaT cells exposed to hydrogel extracts at 24 h measured by CCK‐8 assay. Data are presented as mean ± SEM, and statistical significance was determined using one‐way ANOVA followed by Tukey's post hoc test. *p < 0.05 versus control group. (G) HPLC standard curve for LK‐VIII quantification showing linear regression analysis (R^2^ > 0.99). (H) In vitro cumulative release profile of LK‐VIII from hydrogel over 48 h in PBS and diabetic simulation microenvironment (PBS with 16.6 mM Glucose, 4% BSA and 100 ng/mL MMP‐9) at 37°C. Data are presented as mean ± SEM. (I, J) Skin sensitization test results in mice.

LK‐VIII as a wound healing‐promoting active peptide faces limitations when applied in liquid form, including poor adhesion and rapid degradation, which result in a short drug efficacy duration. To overcome these deficiencies and achieve sustained release of LK‐VIII at wound sites, we developed a thermosensitive PLGA‐PEG‐PLGA triblock copolymer hydrogel system as a drug carrier. The target copolymer was successfully synthesized via melt polycondensation and was confirmed using multiple characterization techniques. Fourier‐transform infrared spectroscopy (FTIR) analysis revealed characteristic absorption peaks at 1749 cm^−1^ (ester carbonyl ─C═O stretching) as well as at 1084 and 1181 cm^−1^ (C─O─C stretching and ─COO stretching vibrations of PEG ‐OCH_2_CH_2_ repeating units), respectively (Figure 7A). Proton nuclear magnetic resonance (^1^H NMR) spectroscopy further verified the copolymer structure (Figure 7B).

Based on the synthesized triblock copolymer, we prepared a 15% (w/v) thermosensitive hydrogel that exhibited pronounced temperature responsiveness: it flowed freely at 4°C but rapidly transformed into a gel state at 37°C (Figure 7C). The hydrogel demonstrated excellent adhesiveness and extensibility, effectively conforming to irregular wound surfaces (Figure 7D; Figure S4B), while allowing barrier‐free injection through a 27‐gauge medical syringe while maintaining shape stability (Figure 7E). Additionally, we conducted more tests on the adhesiveness of the hydrogel to different tissues, including the heart, spleen, kidney, liver, and skin. The results support the conclusion that the gel exhibits good retention properties on the wound surface (Figure S4A).

A biosafety evaluation indicated that the hydrogel leachates showed no toxicity toward HaCaT cells, with cell viability maintained above 100% (Figure 7F). Using HPLC/LCMS technology, we established a standard curve for LK‐VIII (Figure 7G) and examined the in vitro release profile, which demonstrated sustained release for at least 48 h (Figure 7H). To further evaluate the release kinetics of the hydrogel under diabetic conditions, we assessed the drug release profile using PBS buffer supplemented with high glucose and matrix metalloproteinases (MMPs) to mimic the pathological microenvironment. The results indicated that, despite altered release dynamics compared to normal conditions, the LK‐VIII‐loaded hydrogel maintained a sustained release profile at 48 h even within this simulated diabetic environment (Figure 7H). To evaluate drug release in the actual wound, we loaded the hydrogel with a FITC‐labeled short peptide (CGGGGGRGGK‐FITC, OriLeaf, S22213). Fluorescence imaging confirmed that the peptide was effectively retained and released at both 24 and 48 h in the wound area (Figure S4C). These results indicate that the hydrogel maintains sustained release properties even in complex physiological environments. Skin sensitization tests revealed no local irritation reactions (Figure 7I,J), and systemic toxicity evaluation revealed no pathological changes in the major organs (Figure S4D). Furthermore, we conducted additional experiments by administering LK‐VIII to mice and analyzed key immune and allergic markers in the wound tissue and the surrounding skin. We evaluated the infiltration of T cells (CD3^+^) and leukocytes (CD45^+^) through immunohistochemistry (Figure S4E,F). We assessed the presence and degranulation of mast cells, which are primary indicators of allergic responses, using Toluidine Blue staining (Figure S4G). Our data revealed no significant increase in the infiltration of T cells, leukocytes, or mast cells in the LK‐VIII treated group compared to the control group after repeated application. These findings suggest that LK‐VIII elicits negligible immunogenicity and does not trigger significant allergic reactions in mice.

In summary, we successfully constructed an LK‐VIII‐loaded thermosensitive hydrogel system with favorable physicochemical properties and biocompatibility, enabling controlled drug release at wound sites and laying the groundwork for future wound healing research.

### 2.8 LK‐VIII‐Loaded Hydrogel Accelerates Cutaneous Wound Healing in Murine Model

**FIGURE 8:**
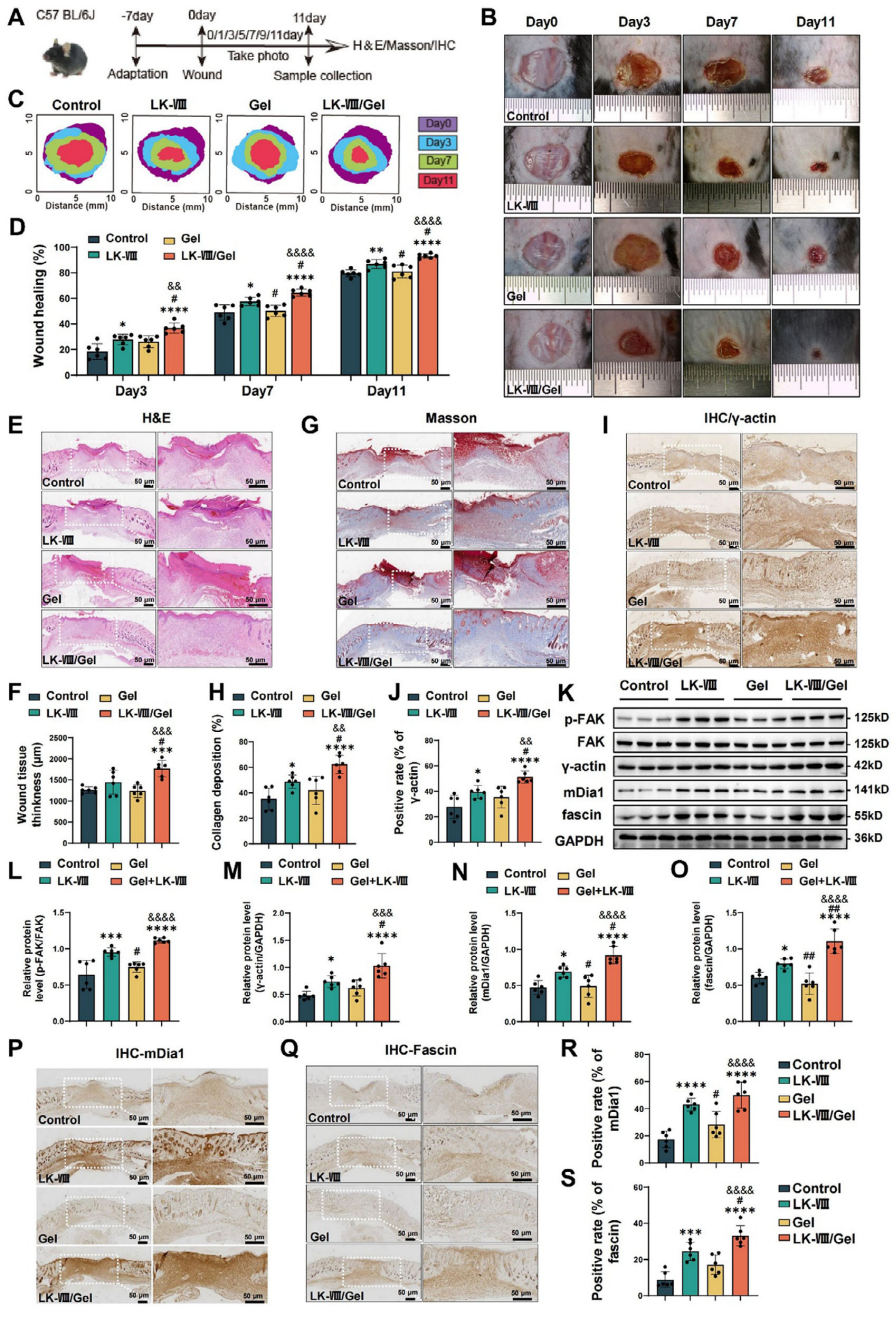
LK‐VIII‐loaded hydrogel enhanced cutaneous wound healing through ACTG1‐mediated cytoskeletal regulation. (A) Schematic illustration of experimental design and timeline for wound healing study in C57BL/6J mice. (B) Representative macroscopic images showing wound healing progression at days 0, 3, 7, and 11 post‐wounding in control, LK‐VIII, Gel and LK‐VIII/Gel groups. (C) Schematic illustration of the wound bed healing process after LK‐VIII and LK‐VIII/Gel treatment. (D) Quantification of wound closure rates over time. (E) H&E‐stained cross sections of wound tissues at day 11. (F) Quantification of wound bed thickness. (G) Masson's trichrome staining showing collagen deposition in wound tissues at day 11. (H) Quantification of collagen‐positive area. (I) Immunohistochemical staining of γ‐actin in wound edge tissues at day 11. (J) Quantification of γ‐actin‐positive area. (K) The protein levels of p‐FAK/FAK, γ‐actin, mDia1 and fascin in skin wound tissues were test by Western blot; GAPDH served as a loading control. (L–O) Densitometric quantification of p‐FAK/FAK ratio (L), γ‐actin (M), mDia1 (N), and fascin (O). Data are presented as mean ± SEM (*n* = 6); (P) Immunohistochemical staining of mDia1 in wound tissues at day 11. (Q) Immunohistochemical staining of fascin in wound tissues at day 11. (R) Quantification of mDia1‐positive area. (S) Quantification of fascin‐positive area. Data are presented as mean ± SEM (*n* = 6). Statistical significance was determined using one‐way ANOVA followed by Tukey's post hoc test. *p < 0.05, **p < 0.01, ***p < 0.001, ****p < 0.0001 versus control group. ^#^ *p* < 0.05,^##^ *p* < 0.01, versus LK‐VIII group. ^&^ *p* < 0.05, ^&&^ *p* < 0.01, ^&&&^ *p* < 0.001, ^&&&&^ *p* < 0.0001 versus Gel group.

To evaluate the therapeutic efficacy of LK‐VIII‐loaded hydrogel in vivo, we employed a standardized full‐thickness excisional wound healing model in C57BL/6J mice. The mice were randomly divided into four groups (*n* = 6): control, free LK‐VIII, hydrogel alone (Gel) and LK‐VIII‐loaded hydrogel (LK‐VIII/Gel, 5 µg/mL). Wound healing progression was monitored for 11 days with sequential photographic documentation, followed by tissue collection for histological analysis (Figure 8A). Macroscopic examination revealed significantly accelerated wound closure in the LK‐VIII‐loaded hydrogel treatment groups compared to the hydrogel control group at 3 (*p* _LK‐VIII/Gel vs Gel_ = 0.0052), 7 (*p*_LK‐VIII/Gel vs Gel_ = 0.0001) and 11 days (*p*_LK‐VIII/Gel vs Gel_ = 0.0039) (Figure 8B). Digital planimetric analysis using wound contour mapping revealed differing wound healing rates among the four groups (Figure 8C). Quantitative assessment revealed that the wound closure rates in the LK‐VIII/Gel group increased by 10.65% at 3 days, 13.76% at 7 days, and 8.40% at 11 days compared to the Gel group (Figure 8D). While the free LK‐VIII treatment also outperformed the control group, the LK‐VIII‐loaded hydrogel exhibited superior efficacy, showing closure rate improvements of 9.11% (*p*_LK‐VIII/Gel vs LK‐VIII_ = 0.018), 6.96% (*p*_LK‐VIII/Gel vs LK‐VIII_ = 0.0472), and 6.23% (*p*_LK‐VIII/Gel vs LK‐VIII_ = 0.0257) over free LK‐VIII at 3, 7, and 11 days, respectively (Figure 8D).

Histological examination revealed that the LK‐VIII/Gel treatment promoted superior tissue architecture compared to both the Gel and free LK‐VIII groups. H&E staining showed complete re‐epithelialization and well‐organized granulation tissue (Figure 8E), while morphometric analysis confirmed a significant increase in wound bed thickness in the LK‐VIII/Gel group (Figure 8F). Consistent with this enhanced regeneration, Masson's trichrome staining demonstrated denser collagen deposition with more orderly fiber alignment (Figure 8G, H). At the molecular level, immunohistochemistry confirmed a significantly higher level of γ‐actin in the LK‐VIII/Gel group versus the Gel group and the free LK‐VIII group (Figure 8I,J). Western blot analyses also revealed a significant upregulation of γ‐actin in the LK‐VIII/Gel group (Figure 8K–O). Moreover, additional immunohistochemical analyses demonstrated an increase in mDia1 and fascin at the wound margins of the LK‐VIII treated group when compared with the Gel group and the free LK‐VIII group (Figure 8P–S). These finding indicate that the LK‐VIII‐loaded hydrogel significantly accelerated wound closure compared to both the hydrogel and free LK‐VIII groups during the skin wound healing process.

Collectively, these macroscopic, histological, and biochemical data indicate that the LK‐VIII‐loaded hydrogel significantly accelerates wound closure in C57BL/6J mice and improves tissue regeneration by enhancing collagen matrix organization, FAK/γ‐actin signaling and filopodia formation, indicating that LK‐VIII has therapeutic potential for promoting wound healing in vivo.

### 2.9 LK‐VIII‐Loaded Hydrogel Promotes Skin Wound Healing in Diabetic Mice Models

To further evaluate the effects of the LK‐VIII peptide on the wound healing process under diabetic conditions, we tested the LK‐VIII‐loaded hydrogel in two established diabetic wound models: streptozotocin (STZ)/high‐fat diet (HFD) treated mice and db/db mice.

**FIGURE 9:**
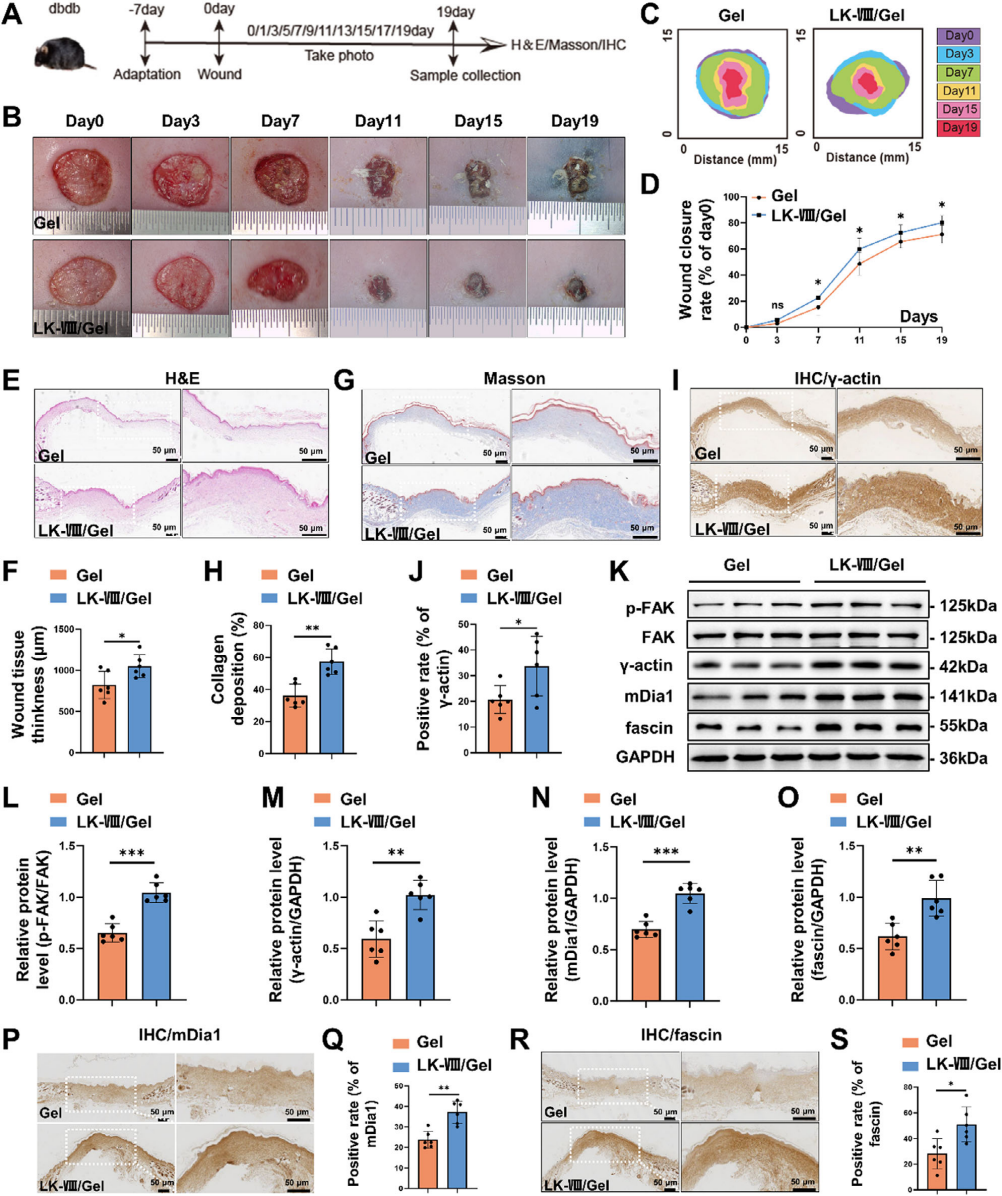
Effects of LK‐VIII‐loaded hydrogel on skin wound healing in db/db diabetic mice. (A) Schematic illustration of experimental design for wound healing study in db/db diabetic mice. (B) Representative macroscopic images of wounds at days 0, 3, 7, 11, 15, and 19 post‐wounding. (C) Schematic illustration of the wound bed healing process. (D) Quantitative analysis of wound closure rates over 19 days. (E) H&E‐stained cross sections of wound tissues at day 19. (F) Quantification of wound bed thickness. (G) Masson's trichrome staining showing collagen deposition at day 19. (H) Quantification of collagen‐positive area. (I) Immunohistochemical staining of γ‐actin in wound tissues at day 19. (J) Quantification of γ‐actin‐positive area. (K) Representative western blot analysis of key signaling proteins in wound tissues. GAPDH served as loading control. (L–O) Densitometric quantification of p‐FAK/FAK ratio (L), γ‐actin (M), mDia1 (N), and fascin (O). Data are presented as mean ± SEM (*n* = 6). (P) Immunohistochemical staining of mDia1 in db/db mouse wound tissues at day 19. (Q) Quantification of mDia1‐positive area in db/db mice. (R) Immunohistochemical staining of fascin in db/db mouse wound tissues at day 19. (S) Quantification of fascin‐positive area in db/db mice. Statistical significance was determined using independent‐sample two‐tailed Student's t‐test; *p < 0.05, **p < 0.01, ***p < 0.001 versus Gel group.

The db/db mice were randomly divided into two groups: hydrogel alone (Gel) and LK‐VIII‐loaded hydrogel (LK‐VIII/Gel) (*n* = 6) (Figure 9A). As expected, db/db mice exhibited significantly delayed healing compared with C57BL/6J mice. Macroscopic analysis demonstrated that LK‐VIII/Gel treatment markedly accelerated wound closure beginning on day 7 (increased by 7.26%, *p*_LK‐VIII/Gel vs Gel_ = 0.0475), with progressively greater differences observed on days 15 (increased by 7.01%, *p*_LK‐VIII/Gel vs Gel_ = 0.0494) and 19 (increased by 10.65%, *p*_LK‐VIII/Gel vs Gel_ = 0.027) compared with Gel group(Figure 9B,C). Quantitative data are presented in Figure 9D. Histologically, LK‐VIII/Gel promoted enhanced tissue regeneration, as evidenced by increased granulation tissue thickness and improved re‐epithelialization (Figure 9E,F). Masson's trichrome staining demonstrated significantly greater collagen deposition with superior fiber organization (Figure 9G,H). Immunohistochemical analysis confirmed elevated γ‐actin level in the LK‐VIII/Gel group (Figure 9I,J). Consistently, western blotting revealed coordinated upregulation of phospho‐FAK, γ‐actin, mDia1, and fascin in LK‐VIII/Gel‐treated wounds (Figure 9K–O), which was further supported by increased mDia1 and fascin staining in wound tissues based on immunohistochemical analysis (Figure 9P–S).

**FIGURE 10:**
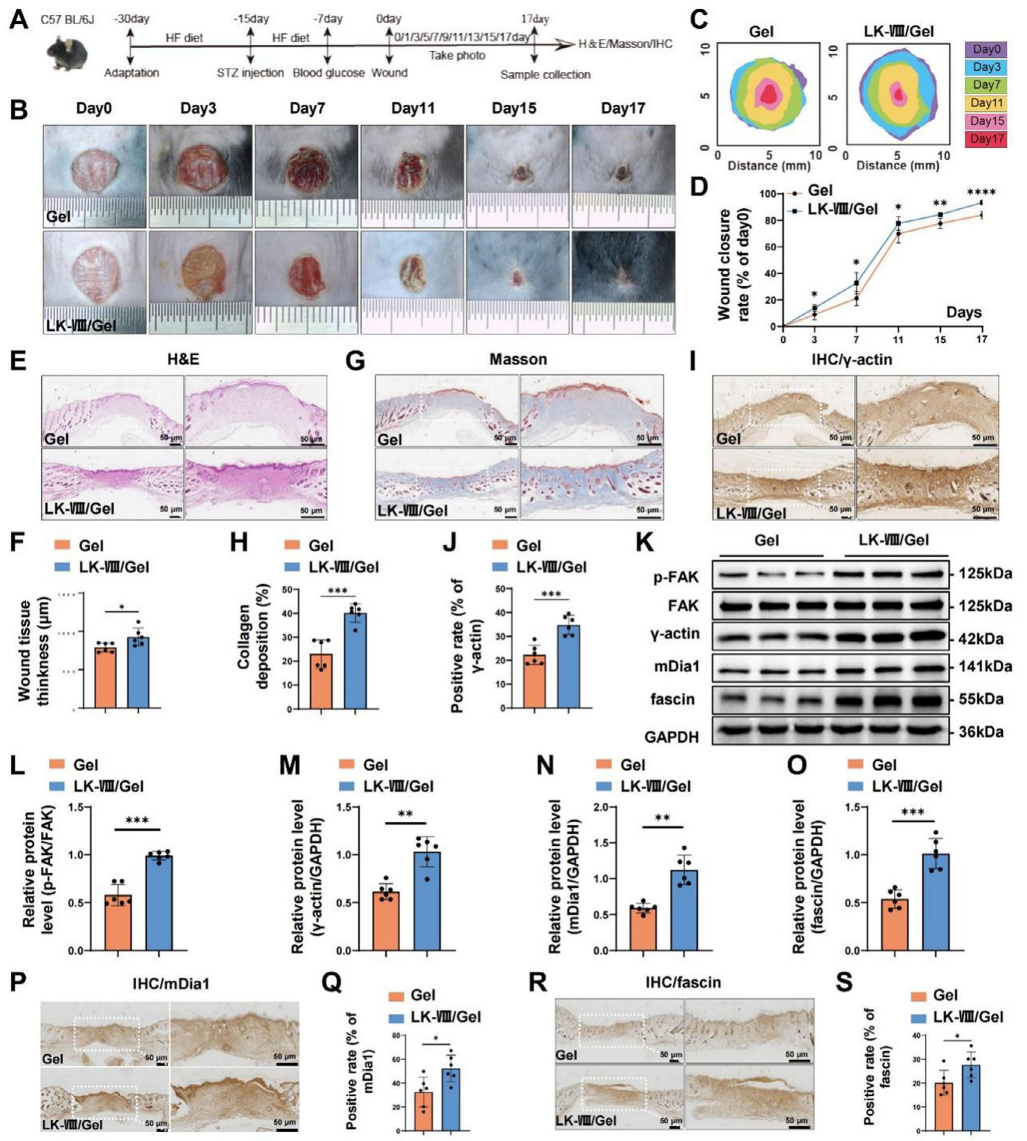
Effects of LK‐VIII‐loaded hydrogel on skin wound healing in STZ/HFD‐induced diabetic mice. (A) Schematic illustration of experimental design for STZ/HFD‐induced diabetes model and wound healing study. (B) Representative macroscopic images of wounds at days 0, 3, 7, 11, 15, and 17 post‐wounding. (C) Schematic illustration of wound bed healing process. (D) Quantitative analysis of wound closure rates over 17 days. (E) H&E‐stained cross sections of wound tissues at day 17. (F) Quantification of wound bed thickness. (G) Masson's trichrome staining showing collagen deposition at day 17. (H) Quantification of collagen‐positive area. (I) Immunohistochemical staining of γ‐actin in wound tissues at day 17. (J) Quantification of γ‐actin‐positive area. (K) Representative western blot analysis of key signaling proteins in wound tissues at day 17. GAPDH served as loading control. (L–O) Densitometric quantification of p‐FAK/FAK ratio (L), γ‐actin (M), mDia1 (N), and fascin (O). Data are presented as mean ± SEM (*n* = 6). (P) Immunohistochemical staining of mDia1 in STZ/HFD‐induced diabetic mouse wound tissues at day 17. (Q) Quantification of mDia1‐positive area in STZ/HFD‐induced diabetic mice. (R) Immunohistochemical staining of fascin in STZ/HFD‐induced diabetic mouse wound tissues at day 17. (S) Quantification of fascin‐positive area in STZ/HFD‐induced diabetic mice. Statistical significance was determined using independent‐sample two‐tailed Student's t‐test; *p < 0.05, **p < 0.01, ***p < 0.001, ****p < 0.0001 versus Gel group.

Subsequently, we assessed the effects of LK‐VIII‐loaded hydrogel on the wound healing process in STZ/HFD‐induced diabetic mice models (Figure 10A). Consistent with the findings in the db/db mice, LK‐VIII/Gel treatment significantly accelerated wound closure relative to hydrogel alone, with notable improvements beginning on day 3 (Figure 10B–D). Histological analysis showed that LK‐VIII/Gel enhanced tissue quality with increased granulation tissue thickness and superior collagen organization in the LK‐VIII/Gel group (Figure 10E–J). At the molecular level, the level of p‐FAK, γ‐actin, mDia1 and fascin were notably increased following LK‐VIII/Gel treatment in wounds of STZ‐treated mice (Figure 10K–O). Immunohistochemistry further validated increased mDia1 and fascin level in LK‐VIII/Gel‐treated wounds (Figure 10P–S).

Together, these results demonstrate that LK‐VIII‐loaded hydrogel effectively promotes wound healing across both diabetic models through consistent activation of FAK‐ACTG1‐mediated filopodia formation, supporting its therapeutic potential for diabetic wound care.

## 3 Discussion

Kangfuxin liquid (KFX), the traditional Chinese medicinal extract derived from the cockroach *P. americana* (Patent: Z51021834), has been widely used in clinical practice for wound healing and tissue repair in China [27, 28]. However, the variability in quality and therapeutic efficacy among different batches and manufacturers has significantly hindered further clinical standardization and broader therapeutic applications. This is also a common challenge faced by Chinese medicines derived from plants and animals whose active compositions remain unclear. Therefore, efforts to discover the active ingredients in the KFX extract and understand how these active ingredients function will significantly enhance the global application of KFX. Polypeptides have recently been recognized as one of the primary active substances that underpin the efficacy of KFX extracts [29]. In the current study, we specifically investigated LK‐VIII, an octapeptide (GASFYSWG‐NH2) isolated from *P. americana*, and demonstrated that LK‐VIII effectively promotes diabetic wound healing by reversing impaired keratinocyte migration (Figure 2E–H). Our in vivo studies employing diabetic mice models (both db/db diabetic mice and STZ‐induced diabetic mice) indicate that the topical application of LK‐VIII significantly accelerates cutaneous wound healing (Figures 9B–D and 10B–D). This finding suggests that LK‐VIII functions as a key active ingredient contributing to the beneficial effects of KFX extracts in promoting wound healing. Additionally, a recent study by Li et al. [30] identified another notable bioactive peptide, PEEPA, which was isolated from the same extract. PEEPA promotes tissue regeneration by activating endogenous stem cells and modulating inflammatory responses, further contributing to skin and tissue repair. Collectively, these findings indicate that bioactive peptides from KFX extracts may function synergistically in a multi‐targeted manner to facilitate tissue regeneration. It remains crucial to thoroughly investigate the specific signaling pathways, detailed mechanisms, and clinical effects of these individual peptides. The systematic isolation, characterization, and validation of the biological activities of individual peptides hold significant potential for clinical translation in regenerative medicine and wound‐healing therapies.

Cytoskeletal remodeling plays a critical role in regulating keratinocyte migration and proliferation [31]. As a key type of cytoskeleton, the dynamic reorganization of actin directly impacts cellular motility [32]. In this study, through transcriptome sequencing, we demonstrated that *ACTG1*, a well‐known actin cytoskeletal component, is a downstream gene following LK‐VIII exposure (Figure 3B,C,E). LK‐VIII significantly upregulated *ACTG1* expression in keratinocytes and skin of diabetic mice, thereby accelerating wound closure, whereas AGEs treatment decreased *ACTG1* expression, ultimately impairing the repair process (Figures 4A–C, 9I–K,M, and 10I–K,M). Notably, silencing *ACTG1* expression by siRNAs weakened LK‐VIII‐mediated keratinocyte migration, confirming that *ACTG1* is critical for skin wound healing (Figure 4F–I). These findings indicate that *ACTG1* plays a pivotal role in mediating the beneficial effects of LK‐VIII on diabetic skin wound healing. The role of *ACTG1* in cell migration has been predominantly documented in cancer cells, as summarized by Suresh [33]. To our knowledge, this is the first study to clarify the role of ACTG1 in keratinocyte function and diabetic wound repair. Moreover, lamellipodia and filopodia in skin fibroblasts and keratinocytes facilitate cell migration, promoting wound healing [34]. *ACTG1* is not only essential for the formation of lamellipodia by localizing at the leading edge and playing an indispensable role in lamellipodial protrusion and cell migration [35], but it is also notably enriched in axonal filopodia, where it stabilizes F‐actin polymers, thereby promoting filopodia extension [36]. Filopodia are thin, actin‐rich plasma membrane protrusions that act as sensory organelles, probe the local environment and establish focal adhesions required for cell traction [37]. In the context of wound healing, keratinocytes at the wound edge undergo a transition to a migratory phenotype. The formation of filopodia is a critical early step in this process, as these structures guide the extension of lamellipodia and drive the directional movement of cells into the wound bed [38]. Previous studies demonstrated that the regulation of actin dynamics, specifically the assembly of filopodia, directly correlates with the velocity and persistence of keratinocyte migration [39]. Therefore, the ability of LK‐VIII to stimulate filopodia formation suggests that it enhances bioactivity by activating the motility machinery of keratinocytes, ultimately facilitating rapid re‐epithelialization. The results from immunostaining and scanning electron microscopy assays showed that LK‐VIII treatment could significantly increase the formation of filopodia in keratinocytes (Figure 5A–C). Following LK‐VIII treatment, the level of mDia1 and fascin, two key markers of filopodial growth [40, 41], was remarkably elevated in cell and animal experiments (Figures 5, 8, 9‐S, 9K,N‐S, and 10K,N‐S). These findings suggest that LK‐VIII‐induced upregulation of *ACTG1* enhances filopodia formation, thereby alleviating diabetes‐induced impairment of skin wound healing. Our data also highlight *ACTG1* as an important molecular target whose modulation can significantly impact cytoskeletal reorganization and filopodia formation and accelerate tissue repair under diabetic conditions, offering a promising therapeutic avenue to improve diabetic skin wound healing.

After KEGG analysis, DEGs derived from transcriptomic data in keratinocytes after LK‐VIII treatment were significantly enriched in signaling pathways such as focal adhesion (Figure 3D). Cell adhesion is essential for cell motility and is mediated by focal adhesion complexes that link the extracellular matrix to the actin cytoskeleton. In moving cells, these focal adhesion complexes are highly dynamic and form near the leading edge, thereby providing mechanical stability and facilitating signal transduction. Among the key signaling pathways involved, FAK signaling promotes the dynamic turnover of focal adhesions, which is essential for cell motility. Subsequently, we further validated that LK‐VIII potently activates the FAK pathway (Figure 6I,J). Given that FAK is an important pathway mediating extracellular matrix‐driven microenvironmental responses, additional experiments using inhibitors demonstrated that FAK regulates *ACTG1* expression, filopodia formation, and is critical for LK‐VIII‐induced keratinocyte migration (Figure 6A–D). To further determine whether FAK signaling mediates the wound healing potential of LK‐VIII, the FAK inhibitor was incorporated into the LK‐VIII‐loaded gel. The results showed that the beneficial effects of the peptide were significantly abrogated in the presence of the inhibitor (Figure S5). This aligns with a previous study by Liu et al. [42] that reported that FAK inactivation under diabetic conditions contributes to impaired skin wound healing. This evidence not only confirms the therapeutic potential of FAK activation in promoting diabetic wound repair but also identifies FAK as a promising target for future therapeutic development.

However, whether LK‐VIII can cross the cell membrane to exert its effects, and the cellular localization of its potential targets is also unknown. We utilized a Membrane and Cytosol Protein Extraction Kit to isolate the cytosolic fraction of HaCaT cells treated with LK‐VIII. Subsequent HPLC/LCMS analysis revealed that LK‐VIII was undetectable in the cytosolic lysates (Figure S6A–C). This suggests that LK‐VIII is unlikely to cross the cell membrane. Furthermore, given that FAK can be activated by ECM components such as integrins [43], the precise ECM components involved in FAK activation by LK‐VIII remain unknown. Molecular docking simulations were performed using AutoDock software to assess the binding affinities of LK‐VIII with integrin subtypes commonly expressed in skin tissue, including ITGA2, ITGA3, ITGA4, ITGA5, ITGA6, ITGA9, ITGAV, ITGB1, ITGB4, ITGB5, and ITGB6. The corresponding docking scores are summarized in Table S1. Among them, ITGA5, ITGAV, and ITGA3 exhibited the highest binding affinities for LK‐VIII (Figure S6D–F), suggesting their potential as primary molecular targets. Collectively, these findings imply that LK‐VIII likely mediates its biological effects through extracellular signaling, specifically by binding to cell surface integrins to activate the downstream FAK pathway, rather than via intracellular entry. However, the precise molecular mechanism warrants further investigation. Investigating this mechanism will provide critical insights and facilitate computational drug design based on the sequence or structure of LK‐VIII, paving the way for more potent bioactive peptides with specific FAK‐activating properties. Additionally, while our experimental validation has confirmed the central role of the FAK pathway, the transcriptomic landscape revealed by RNA‐seq has also highlighted the enrichment of AGE‐RAGE, FOXO signaling pathways and immune‐inflammatory pathways (Figure S3B,C). These findings suggest that LK‐VIII might exert its effects through a multi‐targeted approach. Further in‐depth investigations are required to fully elucidate these specific mechanisms.

To enhance the efficacy of the bioactive peptide LK‐VIII, this study investigated a hydrogel delivery system to address its key challenges, including poor stability, limited bioavailability, and rapid systemic clearance. Responsive hydrogels, particularly thermosensitive variants, demonstrate exceptional potential for wound management because of their unique physicochemical properties and biocompatibility [44]. These systems maintain a moist wound environment and offer injectability and in situ gelation capabilities [45]. Notably, thermosensitive hydrogels that transition from liquid at low temperatures to solid at physiological temperatures enable controlled drug release and conform perfectly to irregular wound geometries, making them ideal carriers for LK‐VIII delivery [46]. By modifying existing literature methods, we rapidly developed thermoresponsive PLGA‐PEG‐PLGA hydrogel copolymers. Successful synthesis was confirmed by NMR and FTIR spectroscopy analyses (Figure 7A,B). Unlike conventional hydrogels with permanent 3D networks and water‐insoluble properties [47], the PLGA‐PEG‐PLGA copolymer demonstrated unique thermoresponsive sol‐gel transition behavior. Below its phase transition temperature, the polymer remains fully soluble in aqueous solutions (Figure 7C), facilitating homogeneous dissolution and structural preservation of LK‐VIII. Upon exposure to physiological temperatures (above the phase transition threshold), the aqueous polymer solution underwent rapid in situ gelation (15–20 s response time, Figure 7C), forming a mechanically robust hydrogel network. This temperature‐triggered phase transition enables dual functionality: (1) efficient peptide encapsulation during gelation via hydrophobic interactions between PLGA blocks and (2) sustained release kinetics mediated by gradual hydrogel erosion at the wound interface. The engineered system exhibits critical clinical advantages: (1) shear‐thinning behavior (evidenced by rheological analysis in Figure 7E) permits smooth extrusion through syringes for conformal coverage of irregular wounds; (2) the gel‐to‐sol transition upon cooling (4°C) allows long‐term storage of LK‐VIII‐loaded formulations without peptide aggregation, addressing cold‐chain logistics challenges; and (3) strong interfacial adhesion ensures prolonged residence time at dynamic wound sites (Figure 7D; Figure S4B). Using this PLGA‐PEG‐PLGA‐based thermosensitive hydrogel, topical application of LK‐VIII accelerated wound closure in both db/db and STZ‐induced diabetic mice models (Figures 9B–D and 10B–D), indicating a successful platform for sustained peptide delivery.

To further enhance the utility and therapeutic efficacy of LK‐VIII in clinical applications, several promising strategies warrant exploration. First, chemical modifications such as PEGylation could significantly improve the stability and extend the half‐life of LK‐VIII in vivo. PEGylation not only protects peptides from rapid enzymatic degradation but also reduces immunogenicity, thereby improving their pharmacokinetics and bioavailability [48]. Second, encapsulating LK‐VIII with extracellular vesicles, such as exosomes derived from skin stem cells, could enhance its absorption efficiency. As natural nanocarriers, exosomes exhibit strong biocompatibility and cell‐targeting capabilities, making them ideal for peptide delivery [49]. Third, the delivery of bioactive peptides via nanotechnology‐based drug delivery systems can address challenges such as poor stability, limited bioavailability, and rapid clearance [50]. The development of advanced nanocarriers allows precise regulation of the release kinetics and tissue distribution of LK‐VIII, thereby improving its therapeutic efficiency. Several growth factors, including epidermal growth factor (EGF) and vascular endothelial growth factor (VEGF) [51], as well as numerous natural products [52], have been shown to effectively promote wound healing. The integration of LK‐VIII with other pro‐healing molecules offers a promising approach to synergistically modulate various stages of the wound repair process. Further development and optimization of these strategies could greatly enhance the clinical potential of LK‐VIII‐loaded hydrogels in diabetic wound treatment.

Despite the promising therapeutic effects of the LK‐VIII peptide observed in this study, several limitations should be acknowledged. First, male mice were predominantly used in our in vivo experiments to minimize the potential confounding effects of the estrous cycle and hormonal fluctuations. Nevertheless, well‐documented evidence [53, 54] indicates significant sex differences in diabetic wound healing. Therefore, further investigations into the therapeutic efficacy and mechanisms of LK‐VIII in female models are necessary to ensure its broad applicability. Second, while the biological activity of LK‐VIII has been demonstrated in this study, its specific receptor or direct binding targets remain unidentified. Clarifying the precise molecular interactions is crucial for optimizing the peptide's design and comprehensively understanding its signaling pathways. Finally, while rodent models offer valuable insights, they do not precisely mimic human skin physiology. Future studies employing large animal models, such as pigs or non‐human primates, which have greater anatomical and physiological similarities to human skin, are essential to validate the safety and efficacy of LK‐VIII before its potential clinical translation.

## 4 Conclusions

*Periplaneta americana*‐derived LK‐VIII is a bioactive peptide that potently promotes keratinocyte migration under diabetic conditions. Mechanistically, LK‐VIII activates the FAK‐ACTG1 signaling axis and induces filopodia formation, thereby enhancing keratinocyte motility. In vivo, LK‐VIII is incorporated into a PLGA‐PEG‐PLGA thermosensitive hydrogel that allows for sustained peptide release, resulting in significantly accelerated wound closure in both STZ/HFD and db/db diabetic mice models. Collectively, this work identifies LK‐VIII as a therapeutic peptide that improves diabetic wound healing via FAK‐ACTG1‐mediated filopodia formation and establishes the PLGA‐PEG‐PLGA thermosensitive hydrogel as a promising translational platform for sustained peptide delivery, offering a potential intervention for diabetic wound management.

## 5 Materials and Methods

### 5.1 Hydrogel Preparation

Following a previously described method [55], polyethylene glycol (PEG1000), D, L‐lactide (DL‐LA), glycolide (GA), and stannous octoate (Sn(Oct)_2_) were added to a single‐necked round‐bottom flask equipped with a magnetic stir bar. The flask was evacuated under vacuum for 30 min and sealed. The reaction vessel was then immersed in an oil bath preheated to a specified temperature. After complete melting of the mixture, the reaction system was vigorously stirred and maintained for 10 h. Upon completion of the reaction, the system was cooled to room temperature and treated with 4°C distilled water under low‐temperature agitation until full dissolution. The resulting mixture was subsequently heated to 70°C to precipitate the polymer. After decanting the supernatant, the purification cycle (dissolution‐precipitation‐decantation) was repeated three times. Finally, the precipitated polymer was freeze‐dried to obtain the hydrogel polymer (PLGA‐PEG‐PLGA).

### 5.2 Structural Characterization of Hydrogel Polymers

The dried PLGA‐PEG‐PLGA polymer sample was prepared as a 10 mg/mL solution in dichloromethane. A 50 µL aliquot of this solution was pipetted and evenly coated onto the surface of a transparent potassium bromide (KBr) window. The coated windows were then air‐dried using an infrared lamp to evaporate the solvent. FTIR spectroscopy was then employed to record the infrared absorption spectrum of the test sample across the wavelength range of 400–4000 cm^−^^1^.

The sample solution was prepared in deuterated chloroform (CDCl_3_, Fluka Chemical; deuteration degree > 99.8%) at a concentration of 15 mg/mL. ^1^H NMR spectra were recorded using a Bruker AV‐500 nuclear magnetic resonance spectrometer operating at 500 MHz.

### 5.3 Gelation and Injectability Assessment

The sol‐gel transition was determined using the vial tilting method at 37°C, with gelation confirmed by the absence of flow. Injectability was assessed qualitatively through manual syringe operation and visual observation of the flow through the needles. All experiments were performed in triplicate.

### 5.4 Cell Culture and Transfection

HaCaT cells (Meisen Cell Technology, Zhejiang, China) were cultured in DMEM supplemented with 10% FBS and 1% penicillin/streptomycin at 37°C with 5% CO_2_. Transfections were performed using jetPRIME reagent (Polyplus) with sh‐Ctrl or *ACTG1*‐targeting shRNA vectors (MiaoLingBio) according to the manufacturer's protocols (2 µg plasmid, 4 µL jetPRIME, 200 µL buffer per well).

### 5.5 CCK‐8

HaCaT cells were seeded in 96‐well plates (5 × 10^3^ cells/well) and treated for 24 h. Cell viability was assessed using CCK‐8 reagent (APExBIO). After treatment, 10 µL CCK‐8 reagent in 90 µL serum‐free medium was added, incubated for 1 h at 37°C and absorbance was measured at 450 nm. All experiments were performed in triplicate.

### 5.6 Scratch Wound Healing Assay

HaCaT cells were seeded in 6‐well plates (2 × 10^5^ cells/well) until 80%–90% confluent. Linear scratches were created using 200 µL pipette tips, debris was removed by PBS washing, and serum‐free medium was added. Wound healing was photographed at 0, 12, 24, 36, and 48 h using an inverted microscope and quantified using the ImageJ software. Three independent experiments were performed.

### 5.7 Transwell Migration Assay

Cell migration was assessed using Transwell chambers. HaCaT cells (3 × 10^4^ cells in 200 µL serum‐free DMEM) were seeded in upper chambers, with 600 µL complete DMEM in lower chambers as a chemoattractant. After 24 h at 37°C, cells were fixed with 4% paraformaldehyde, stained with crystal violet, and non‐migrated cells were removed. Migrated cells were counted in five random fields per sample using ImageJ software. All experiments were performed in triplicate.

### 5.8 Immunofluorescence Staining

The cells were fixed with 4% paraformaldehyde (15 min), permeabilized with 0.1% Triton X‐100 (10 min), and blocked with 5% BSA (1 h). F‐actin was visualized using fluorescein isothiocyanate‐phalloidin (Biosharp, 1:1000, 60 min). For immunofluorescence, cells were incubated with anti‐γ‐actin antibody (Abcam, ab123034, 1:500) overnight at 4°C, followed by a fluorophore‐conjugated secondary antibody (Proteintech, RGAR003, 1:1000). Nuclei were counterstained with DAPI and imaged using an inverted fluorescence microscope (Olympus, Tokyo, Japan).

### 5.9 RNA Sequencing (RNA‐seq) Analysis

Total RNA from LK‐VIII‐treated HaCaT cells was extracted using the TRIzol reagent. Following quality assessment, high‐throughput sequencing was performed using the Illumina NovaSeq 6000 platform (Shanghai Meiji Biotech). Differentially expressed genes (DEGs) were identified using DESeq2 (v1.38.0) at FDR < 0.05 and |log_2_FC| ≥ 0.585. GO and KEGG enrichment analyses were performed using clusterProfiler (v4.6.0) with Fisher's exact test and Benjamini‐Hochberg correction (adjusted *p* < 0.05, gene set size ≥ 10). GSEA was conducted using GSEA v4.3.2 on the DESeq2 Wald statistic‐ranked gene list against MSigDB v2023.1 Hs (Hallmark, C2, C5 and C7 collections) with the following parameters: gene set size of 15‐500, 1000 permutations, and FDR < 0.25. PPI networks were constructed using STRING (v12.0, confidence ≥ 0.7) and analyzed in Cytoscape (v3.9.1) with the CytoNCA plugin (v2.1.6). Hub genes were defined as nodes with degree ≥ 2‐fold median, and functional modules were identified using MCODE (v2.0.0). The data were deposited at CNCB (https://www.cncb.ac.cn/) under the accession number PRJCA047944.

### 5.10 Animals

Male C57BL/6J mice (6–8 weeks), db/db mice (10 weeks old), and HFD‐fed STZ‐induced diabetic mice were obtained from Sipeifu Biotechnology (Beijing, China; License SCXK‐2024‐0001). The animals were housed under SPF conditions (22‐26°C, 40%–60% humidity, 12 h light/dark cycle) with ad libitum access to food and water. All procedures were approved by the Southwest Medical University Animal Ethics Committee (SWMU20250015).

### 5.11 STZ/HFD‐Induced Diabetes Model

Six‐week‐old male C57BL/6J mice were fed a HFD for 4 weeks to induce insulin resistance. After an overnight fast (12 h), STZ (50 mg/kg) was freshly dissolved in sodium citrate buffer (0.1 mol/L, pH 4.5) and administered intraperitoneally within 15 min. The mice continued to receive a HFD for an additional 4 weeks following STZ injection to maintain insulin resistance. Successful induction of type 2 diabetes was confirmed by fasting blood glucose levels > 11.1 mmol/L on two consecutive readings taken at 72 h post‐injection. Blood glucose levels were monitored weekly using a glucometer. Only mice meeting the diabetic criteria were included in the subsequent wound healing experiments.

### 5.12 Full‐Thickness Wound Model

Mice were anesthetized with inhaled isoflurane followed by an intraperitoneal injection (0.2 mL/10 g body weight), and placed in a prone position. The dorsal hair was shaved, and the skin was disinfected with 75% ethanol, followed by excision of the full‐thickness skin along the dorsal midline using an 8‐mm diameter ophthalmic trephine to create standardized circular wounds. Post‐procedure, the mice were placed in a 37°C environment for recovery, with close monitoring of vital signs for 6 h after awakening. The day of model creation was designated as Day 0. Wound images were captured using a digital surgical microscope (Reward, China) at different time points depending on the healing rate of each model: 0, 3, 7, and 11 days for wild‐type C57BL/6J mice: 0, 3, 7, 11, 15, and 17 days for STZ/HFD‐induced diabetic mice; and 0, 3, 7, 11, 15, and 19 days for db/db diabetic mice. An extended observation period for diabetic models was necessary due to delayed wound healing compared to wild‐type mice. Wound areas were measured using ImageJ software, and healing rates were calculated and analyzed using GraphPad Prism.

### 5.13 In Vivo Skin Irritation Test

Skin irritation tests were conducted according to the method reported by Fang et al. [56] Healthy male C57BL/6J mice were anesthetized, their dorsal hair was shaved, and the skin was disinfected. The dorsal area was divided into left and right testing regions to evaluate: blank hydrogel versus LK‐VIII (5 µg/mL)‐loaded hydrogel. The hydrogels were secured with dressings, and the treated sites were photographed at 0 and 24 h. At least three mice were used per group, and irritation responses were assessed.

### 5.14 Western Blot

Total protein was extracted using RIPA buffer and quantified using a bicinchoninic acid assay. Proteins (20 µg) were separated by 10% sodium dodecyl sulfate‐polyacrylamide gel electrophoresis and transferred onto nitrocellulose membranes. After blocking with 5% non‐fat milk, membranes were incubated overnight at 4°C with primary antibodies. γ‐actin (Abcam ab123034, 1:1000), E‐cadherin (Beyotime AF1552, 1:1000), N‐cadherin (Beyotime AG1554, 1:1000). Vimentin (Beyotime AF1975, 1:1000), GAPDH (Proteintech 10494‐1‐AP, 1:5000), p‐FAK (CST #3283, 1:1000), FAK (CST #3285, 1:1000), mDia1 (Proteintech 20624‐1‐AP, 1:1000), and fascin (Proteintech 14384‐1‐AP, 1:1000) were used, and HRP‐conjugated secondary antibodies (Proteintech, 1:5000) were applied for 2 h. Proteins were detected using ECL reagent (Biosharp BL520A), quantified with ImageJ, and normalized to GAPDH.

### 5.15 Histological and Immunohistochemical Analysis

Mice wound tissues with 5 mm surrounding skin were fixed in 4% paraformaldehyde for 24 h, dehydrated through graded ethanol, embedded in paraffin, and sectioned to a thickness of 4 µm. H&E staining was performed following standard protocols to assess tissue morphology, and Masson's trichrome staining was conducted using a commercial kit (Leagene, DC0033) according to the manufacturer's instructions to evaluate collagen deposition. For immunohistochemistry, tissue sections were deparaffinized, rehydrated, and subjected to antigen retrieval. Endogenous peroxidase activity was blocked with 3% hydrogen peroxide, and non‐specific binding was blocked with 5% goat serum for 1 h at room temperature. The sections were then incubated with specific primary antibodies overnight at 4°C, washed with PBS, and incubated with horseradish peroxidase‐conjugated secondary antibodies for 30 min at room temperature. Immunoreactivity was visualized using DAB as the chromogen, followed by hematoxylin counterstaining. High‐resolution images were acquired using a digital slide scanner for analysis.

### 5.16 Statistical Analysis

Statistical analyses were performed using SPSS (v23.0) and GraphPad Prism (v10.1.2). All quantitative data are presented as mean ± standard deviation (SD), with each experimental group containing a minimum of three independent biological replicates. Comparisons between two groups were evaluated using independent‐sample two‐tailed Student's t‐test, whereas multiple group comparisons were conducted using one‐way analysis of variance (ANOVA) followed by Tukey's post‐hoc test.

## Author Contributions

Z.Q., J.W., and X.X. contributed to the study design, experimental implementation, data analysis, and manuscript preparation. X.Z. contributed to the methodology development and experimental protocol optimization. X.W. and N.D. contributed to the formal analysis and data interpretation. L.M. and J.L. undertook a substantial amount of work during the investigation phase. L.M. contributed to the data visualization. C.O. contributed to the critical discussion and data presentation. J.F. and N.M. conceived and supervised the research, provided funding support, and critically revised the manuscript. All authors contributed to the manuscript and approved the final version.

## Funding

This work was supported by Sichuan Science and Technology Program (No. 2025ZNSFSC0744), Health Commission of Sichuan Province Medical Science and Technology Program (Nos. 24QNMP040, 25QNMP087), and Luzhou Science and Technology Program (No. 2025RCX002).

## Conflicts of Interest

The authors declare that they have no competing interests. A patent application related to this work was filed by The Affiliated Hospital, Southwest Medical University (CN202411858098.4).

## Supporting information




**Supporting File**: advs74459‐sup‐0001‐SuppMat.docx.

## Data Availability

The data that support the findings of this study are available from the corresponding author upon reasonable request.
