# Supplementary material for: Cockroach‐Derived Leucokinin VIII Peptide Accelerates Diabetic Skin Wound Healing by Enhancing Keratinocyte Filopodia Formation
Source: Adv Sci (Weinh). 2026 Feb 19;13(24):e22333. doi: 10.1002/advs.202522333 (PMC13116339; doi:10.1002/advs.202522333)

**Supplementary Figures and Tables**


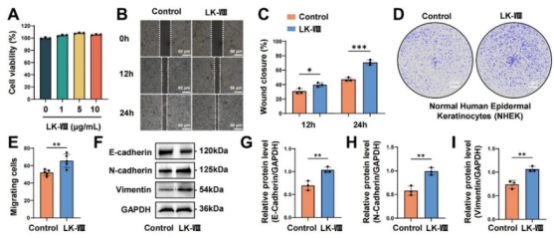


**Supplementary Figure 1.** LK-Ⅷ promoted NHEK cell migration. (A) CCK-8 assay showing the effects of different LK-Ⅷ concentrations (0, 1, 5, 10 μg/mL) on keratinocyte cell viability after 24 h treatment. Data are presented as mean ± SEM. (B) The effects of LK-Ⅷ (5 μg/mL) on NHEK cell migration was examined using scratch wound healing assays at 12, and 24 h. (C) Quantification of wound closure percentage. (D) Transwell migration assay was used to demonstrate the effect of LK-Ⅷ (5 μg/mL) on NHEK cell migration. (E) Quantification of migrated cells in five randomly selected microscopic fields (100× magnification). (F) The protein levels of E-cadherin, N-cadherin and Vimentin were examined by Western blot in NHEK cells after 24 h of treatment with LK-Ⅷ (5 μg/mL). GAPDH served as a loading control. (G-I) Densitometric quantification of the protein levels of E-cadherin, N-cadherin, and Vimentin was performed, and these levels were normalized to those of GAPDH. **P* < 0.05, ****P* < 0.001 versus control group. Data are presented as mean ± SEM. Statistical significance was determined using independent-sample two-tailed Student's *t*-test.


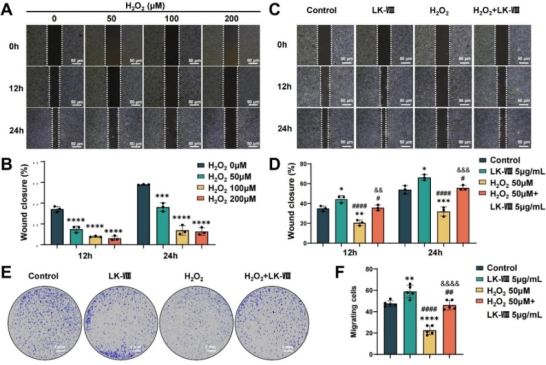


**Supplementary Figure 2.** The protective effects of LK-Ⅷ against H₂O₂ - induced oxidative stress. (A) The effects of different H₂O₂ concentrations (0, 50, 100, and 200 μM) on HaCaT cell migration were examined using scratch wound healing assays at 0, 12, and 24 h. (B) Quantification of wound closure percentage. (C) Scratch assays were performed to evaluate the protective effects of LK-Ⅷ (5 μg/mL) against H₂O₂ (50 μM)-induced inhibition of cell migration over 24 h. (D) Quantification of wound closure percentage at 12 and 24 h. (E) Transwell migration assays showing cell migration in groups treated with LK-Ⅷ, H₂O₂, or their combination. (F) Quantification of migrated cells in Transwell assays for all treatment groups. Data are presented as mean ± SEM. Statistical significance was determined using one-way ANOVA test, followed by Tukey post hoc test.


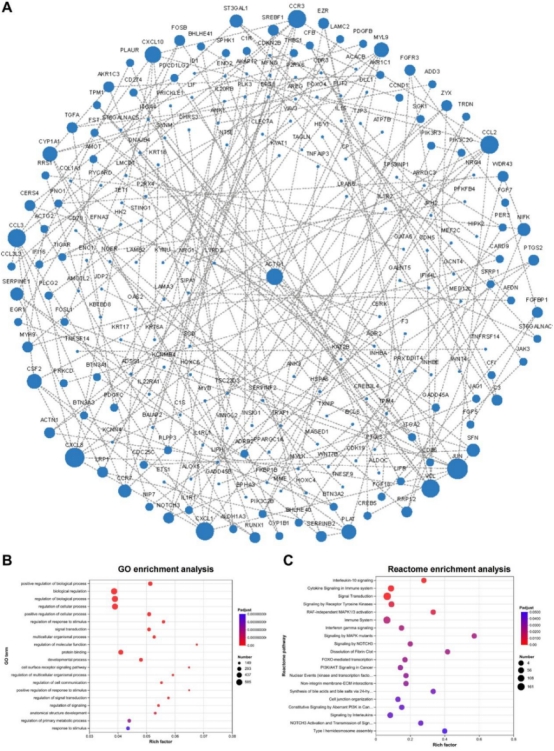


**Supplementary Figure 3.** Protein-protein interaction (PPI) network and functional enrichment analysis of differentially expressed genes. (A) PPI network of DEG-encoded proteins, with node size representing degree centrality and color indicating expression change. ACTG1 occupies a central hub position with extensive connections to cytoskeletal and adhesion-related proteins. (B) Gene Ontology (GO) biological process enrichment analysis. (C) Reactome pathway enrichment analysis.


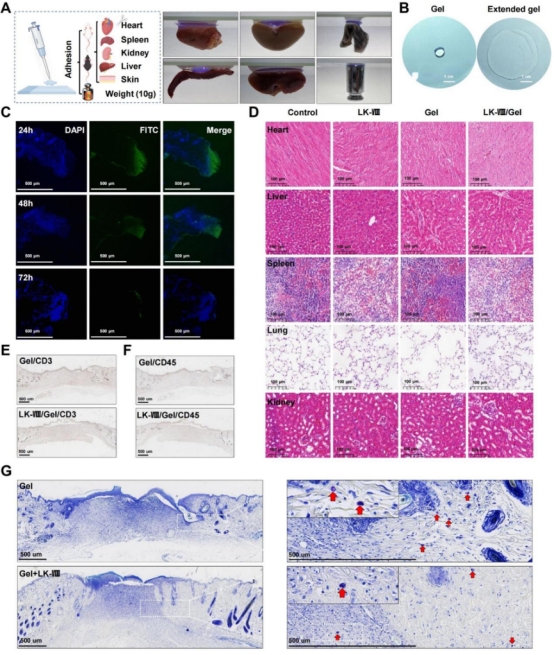


**Supplementary Figure 4.** Adhesiveness, spreadability, and systemic safety evaluation of LK-Ⅷ-loaded hydrogel. (A) Indirect determination of hydrogel adhesion properties includes the ability to vertically adhere mouse heart, spleen, liver, skin, and a 10 g weight onto a glass slide. (B) Spreading test demonstrating hydrogel distribution at 25°C. Scale bar, 1 cm. (C) Fluorescence signal of a FITC-labeled short peptide-loaded hydrogel in the skin wound at 24, 48, and 72 hours. (D) Representative H&E staining of major organs showing no treatment-related histopathological changes in heart, liver, spleen, lung, and kidney tissues. (E, F) Immunohistochemical staining of CD3 (E) and CD45 (F) in the wound edge tissues of mice treated with Gel and LK-Ⅷ/Gel. (G) Toluidine blue staining of mast cells in wound edge tissues of mice treated with Gel or LK-Ⅷ/Gel.


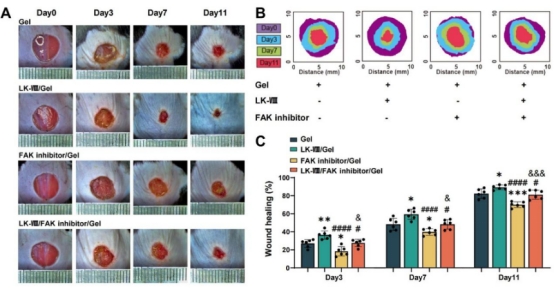


**Supplementary Figure 5.** FAK inhibitor effectively suppressed the promoting effects of LK-Ⅷ on skin wound healing.

(A) The FAK inhibitor PF-573228 was used to treat skin wound healing to assess its impact on the promoting effects of LK-Ⅷ/Gel. Representative macroscopic images showing wound healing progression at days 0, 3, 7, and 11 post-wounding in the Gel, LK-Ⅷ/Gel, FAK inhibitor/Gel and LK-Ⅷ/FAK inhibitor/Gel groups. (B) Schematic illustration of the wound bed healing process after FAK inhibitor and LK-Ⅷ/Gel treatment. (C) Quantification of wound closure rates over time among the Gel, LK-Ⅷ/Gel, FAK inhibitor/Gel and LK-Ⅷ/FAK inhibitor/Gel groups. Data are presented as mean ± SEM. Statistical significance was determined using [independent-sample two-tailed Student's t-test](file:///D:\xwechat_files\wxid_7974ls2hryco21_00d2\temp\RWTemp\2026-01\6762f30748efcd9895c11b2e05e31285\9a01d80fc32c8a243a20669b6dd56aa6.png). **P* < 0.05, ***P* < 0.01, ****P* < 0.001 versus Gel group. *^#^ P* < 0.05, *^####^ P* < 0.0001, versus LK-Ⅷ/Gel. ^&^*P* < 0.05, ^&&&^*P* < 0.001 versus FAK inhibitor/Gel group.


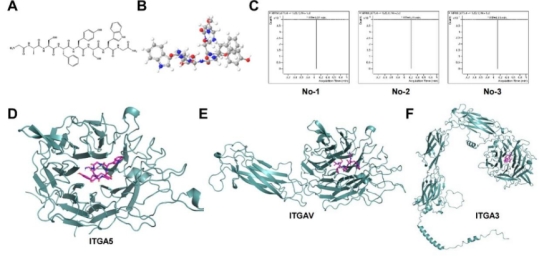


**Supplementary Figure 6. Molecular docking analysis of the interaction between LK-Ⅷ and integrins.** (A) The chemical formula of LK-Ⅷ. (B) Schematic illustration of the peptide structure of LK-Ⅷ. (C) Intracellular localization analysis of LK-Ⅷ. The cytosolic fraction of HaCaT cells treated with LK-Ⅷ was isolated using a Membrane and Cytosol Protein Extraction Kit, and subsequent HPLC/LCMS analysis detected no LK-Ⅷ in the cytosolic lysates, suggesting it does not enter the cytoplasm. (D–F) Molecular docking simulations of LK-Ⅷ with integrin subtypes. AutoDock software was used to calculate binding affinities. Among the subtypes commonly expressed in skin tissue, ITGA5 (D), ITGAV (E), and ITGA3 (F) exhibited the highest binding affinities for LK-Ⅷ.

**Supplementary Table 1**. Summary of molecular docking scores between LK-Ⅷ and various integrin subtypes.


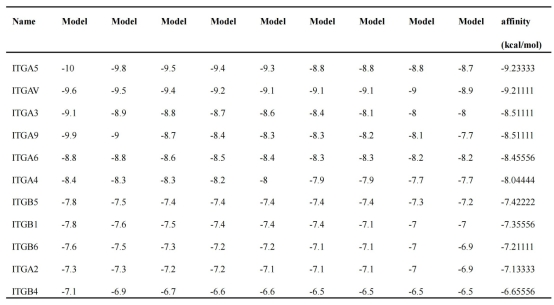

Supplement: Supplementary file 1 — Supporting File: advs74459‐sup‐0001‐SuppMat.docx. [file ADVS-13-e22333-s001.docx]
